# Behavioral and Neurochemical Phenotyping of Mice Incapable of Homer1a Induction

**DOI:** 10.3389/fnbeh.2017.00208

**Published:** 2017-11-01

**Authors:** Michael C. Datko, Jia-Hua Hu, Melanie Williams, Cindy M. Reyes, Kevin D. Lominac, Georg von Jonquieres, Matthias Klugmann, Paul F. Worley, Karen K. Szumlinski

**Affiliations:** ^1^Department of Psychological and Brain Sciences, University of California, Santa Barbara, Santa Barbara, CA, United States; ^2^Department of Neuroscience, Johns Hopkins University School of Medicine, Baltimore, MD, United States; ^3^Department of Molecular, Developmental and Cell Biology, University of California, Santa Barbara, Santa Barbara, CA, United States; ^4^Translational Neuroscience Facility, School of Medical Sciences, University of New South Wales, Sydney, NSW, Australia; ^5^Neuroscience Research Institute, University of California, Santa Barbara, Santa Barbara, CA, United States

**Keywords:** Homer1a, cocaine, glutamate, dopamine, nucleus accumbens, anxiety, sensitization

## Abstract

Immediate early and constitutively expressed products of the *Homer1* gene regulate the functional assembly of post-synaptic density proteins at glutamatergic synapses to influence excitatory neurotransmission and synaptic plasticity. Earlier studies of *Homer1* gene knock-out (KO) mice indicated active, but distinct, roles for IEG and constitutively expressed *Homer1* gene products in regulating cognitive, emotional, motivational and sensorimotor processing, as well as behavioral and neurochemical sensitivity to cocaine. More recent characterization of transgenic mice engineered to prevent generation of the IEG form (a.k.a *Homer1a* KO) pose a critical role for Homer1a in cocaine-induced behavioral and neurochemical sensitization of relevance to drug addiction and related neuropsychiatric disorders. Here, we extend our characterization of the *Homer1a* KO mouse and report a modest pro-depressant phenotype, but no deleterious effects of the KO upon spatial learning/memory, prepulse inhibition, or cocaine-induced place-conditioning. As we reported previously, *Homer1a* KO mice did not develop cocaine-induced behavioral or neurochemical sensitization within the nucleus accumbens; however, virus-mediated Homer1a over-expression within the nucleus accumbens reversed the sensitization phenotype of KO mice. We also report several neurochemical abnormalities within the nucleus accumbens of *Homer1a* KO mice that include: elevated basal dopamine and reduced basal glutamate content, Group1 mGluR agonist-induced glutamate release and high K^+^-stimulated release of dopamine and glutamate within this region. Many of the neurochemical anomalies exhibited by *Homer1a* KO mice are recapitulated upon deletion of the entire *Homer1* gene; however, *Homer1* deletion did not affect NAC dopamine or alter K^+^-stimulated neurotransmitter release within this region. These data show that the selective deletion of Homer1a produces a behavioral and neurochemical phenotype that is distinguishable from that produced by deletion of the entire *Homer1* gene. Moreover, the data indicate a specific role for Homer1a in regulating cocaine-induced behavioral and neurochemical sensitization of potential relevance to the psychotogenic properties of this drug.

## 1 Introduction

The mammalian gene *Homer1* possesses 10 exons, the regulated transcription of which results in both constitutively expressed and immediate early gene (IEG) products (Brakeman et al., 1997; Kato et al., 1998; Bottai et al., 2002). Exons 1–5 encode an Enabled/vasodilator-stimulated phosphoprotein (Vasp) homology 1 (EVH1) domain (Beneken et al., 2000). This EVH1 domain exhibits a RxxxxxGLGF sequence that enables Homer interactions with a proline-rich sequence (PPSPF) displayed by proteins regulating neuronal morphology, synaptic architecture, and glutamate receptor signaling/intracellular calcium dynamics (Shiraishi-Yamaguchi and Furuichi, 2007; Worley et al., 2007; Szumlinski et al., 2008a; Fagni, 2012; cf. Constantin, 2016). Exons 6–10 encode the carboxyl-tail of Homer proteins that consists of a coiled-coil (CC) domain, two leucine zipper motifs, and the 3′ UTR (Kato et al., 1998; Xiao et al., 1998; Soloviev et al., 2000). This CC-domain enables Homer proteins to tetramerize (Hayashi et al., 2006) – a property necessary for maintaining the functional architecture of glutamate synapses (for reviews, Shiraishi-Yamaguchi and Furuichi, 2007; Worley et al., 2007; Szumlinski et al., 2008a; Fagni, 2012; Constantin, 2016).

Alternative transcript splicing in regions downstream from exon 5 and premature termination of gene transcription has been reported for all three *Homer* genes (*Homer1*, *Homer2*, *Homer3*) (Soloviev et al., 2000). However, this phenomenon has been best-described for the *Homer1* gene (Brakeman et al., 1997; Bottai et al., 2002). The premature termination of *Homer1* transcription results in truncated or “short” Homer1 isoforms, so named because they lack the CC- and leucine zipper motifs necessary to multimerize, of which Homer1a and ania-3 have been identified in brain (Brakeman et al., 1997; Berke et al., 1998; Soloviev et al., 2000). The induction of these IEG Homer1 isoforms (i.e., Homer1a and ania-3) upon synaptic activity serves a dominant negative function by displacing CC-Homer proteins from their EVH1-bound partners. While this dynamic expression is predicted to cause a temporary reduction in the efficacy of post-synaptic glutamate transmission, by virtue of their inability to tetramerize, IEG Homer induction enables the trafficking to, and lateral movement within, the plasma membrane of glutamate receptors and associated signaling molecules. Thus, the induction of IEG Homer isoforms is critical for synaptic rearrangement and neuroplasticity (for reviews, Shiraishi-Yamaguchi and Furuichi, 2007; Fagni, 2012; Marton et al., 2015).

Converging evidence from clinical and behavioral/neural genetic studies in animals support active roles for members of the Homer family of post-synaptic scaffolding proteins in regulating various aspects of behavior, as well as drug-induced neuroplasticity relevant to addiction (for reviews, Szumlinski et al., 2008a; Marton et al., 2015). IEG Homer isoforms are induced in mesocorticolimbic structures by acute treatment with various drugs of abuse (see Szumlinski et al., 2008a; Marton et al., 2015). However, our understanding of their functional significance for addiction-related behavioral and neuropathology is rather limited. Earlier virus-mediated gene transfer studies failed to detect significant effects of Homer1a over-expression upon cocaine-induced locomotor hyperactivity/sensitization in rodent models (Lominac et al., 2005; Szumlinski et al., 2006). In contrast, behavioral phenotyping of transgenic mice over-expressing Homer1a selectively within striatal striosomes supported an active role for Homer1a in acute amphetamine-elicited locomotion, as well as in the expression of anxiety (Tappe and Kuner, 2006). The behavioral phenotype of striatal Homer1a over-expressing mice is consistent with those reported previously for *Homer1* whole-gene deletion (Szumlinski et al., 2004, 2005a; Lominac et al., 2005), as well as for rats infused intra-accumbens with oligonucleotides against *Homer1* (Ghasemzadeh et al., 2003), providing support for a competitive interaction between IEG and CC-Homer1 isoforms in regulating addiction-related emotional and motivational processing (Marton et al., 2015).

Herein, we provide a more complete behavioral and neurochemical phenotyping of a transgenic mouse engineered to prevent expression of Homer1a (*Homer1a^-/-^*) while retaining expression of Homer1c (Hu et al., 2010). *Homer1a^-/-^* mice do not exhibit cocaine-induced locomotor or neurochemical sensitization within the NAC (Park et al., 2013). Here, we relate this resiliency to the dysregulation of NAC extracellular dopamine and glutamate and, demonstrate, using an adeno-associated virus (AAV) strategy, an active role for Homer1a induction within the NAC in regulating cocaine-induced neuroplasticity of relevance to addiction and related mental disorders.

## 2 Materials and Methods

### 2.1 Subjects

*Homer1a^+/+^*, *Homer1a^+/-^*, and *Homer1a^-/-^* mice (on a mixed C57BL/6J × 129Xi/SvJ background; see Hu et al., 2010 for details), as well as *Homer1^+/+^* and *Homer1^-/-^* mice (on a mixed BALB/cJ × C57BL/6J × 129Xi/SvJ background; see Yuan et al., 2003 for details) were bred in-house at UCSB from mating of heterozygous breeder pairs and both male and female littermates were employed in all studies. The primers for genotyping *Homer1a^-/-^* mice are: H1F: CACCCGATGTGACACAGAAC, H1cR: CCAGTAATGCCACGGTACG, H1aR298: CACTGCTTCACATTGGCAGT. Wild-type band (H1F/H1cR) is at 602 bp and KO band (H1F/H1aR298) is at 211 bp. The primers for genotyping *Homer1^-/-^* mice are: 84: CAATGCATGCAATTCCTGAG, 85: CGAGAAACTTACATATATCCGCAAA, 86: GAACTTCGCGCTATAACTTCG. Wild-type band (H1F/H1cR) is at 602 bp and KO band (H1F/H1aR298) is at 211 bp. To reduce litter confounds, mice were selected from a minimum of four different litters within each replicate and testing began when mice were 7–8 weeks of age. Animals were group-housed in polyethylene cages in a temperature (25°) and humidity (71%) controlled vivarium under a 12 h dark/light cycle (lights off: 7:00 A.M.) with food and water available *ad libitum*. Experimental protocols, as well as housing and animal care, were consistent with the guidelines provided by the National Institute of Health (NIH) Guide for Care and Use of Laboratory Animals. All experiments were approved by the Institutional Animal Care and Use Committee of University of California, Santa Barbara.

### 2.2 Behavioral Test Battery

Male and female *Homer1a^+/+^*, *Homer1a^+/-^*, *Homer1a^-/-^* littermates were subjected to a behavioral test battery consisting of various conventional paradigms to assay anxiety-like behavior (elevated plus maze, reactivity to a novel object), depressive-like behavior (Porsolt swim test, saccharin preference), spatial learning and memory (Morris water maze), and sensorimotor processing/gating (acoustic startle and pre-pulse inhibition of acoustic startle/PPI). With the exception of the saccharin preference test, the procedures for each of these paradigms are detailed in our published work (see Szumlinski et al., 2004, 2005a; Lominac et al., 2005; Ary et al., 2013; Lee et al., 2015; Guo et al., 2016).

In brief, for the elevated plus maze, mice were placed on the center intersection of the maze with two white open arms and two black-walled arms 24 cm high. Each arm measured 123 cm long by 5 cm wide. Behavior in the apparatus was monitored for a 5 min trial (Lee et al., 2015; Guo et al., 2016). For the reactivity to a novel object test, mice were placed into a black Plexiglas (40 cm × 40 cm × 30 cm) open-field arena, containing an inedible object in the center. Mice were allowed to explore the arena for 2 min and the amount of time spent investigating the object, as well as the number of object contacts were recorded (Ary et al., 2013; Lee et al., 2015). For the Porsolt swim test, mice were placed into Plexiglas buckets (23 cm × 24 cm × 22 cm) and the behavior of the mice was scored in 30-s intervals using a checklist for swimming or floating over a 15-min period (Lominac et al., 2005; Szumlinski et al., 2005a; Ary et al., 2013; Guo et al., 2016). For the saccharin preference test, mice were presented with two 50 ml sipper tubes, one containing tap water and the other containing a 0.16% w/v saccharin solution for 24 h in the home cage. Bottle weights before and after the 24-h drinking period gauged the volume consumed and were used to calculate the preference for the saccharin solution. For the Morris water maze, we employed a stainless steel circular tank (200 cm in diameter, 60 cm in height; painted white on the inside and filled with room temperature water to a depth of 40 cm), with salient extra maze cues located on all four walls of the room in which the maze was located. A clear platform was placed in the tank and its location remained fixed throughout the course of the experiment. For 4 days, mice were trained four times a day (once at each compass point) to locate the hidden platform. During each trial, mice were randomly placed in the pool at one of the four compass points and swimming was recorded digitally by a video camera mounted on the ceiling directly above the pool (ANY-Maze, Stoelting). Training sessions were 120 s in duration; if the mice were unable to locate the platform during the allotted time, they were guided to the platform where they remained for 30 s. At 24 h after the last training trial, a 120-s memory probe test was performed in which the platform was removed and the amount of time taken by the mouse to swim toward the platform location and the time spent swimming in the platform quadrant was recorded (Lominac et al., 2005; Ary et al., 2013). For the acoustic startle/PPI experiment, we presented six different trial types: startle pulse (st110, 110 dB/40 ms), low prepulse stimulus given alone (st74, 74 dB/20 ms), high prepulse stimulus given alone (st90, 90 dB/20 ms), st74 or st90 given 100 ms before the onset of the startle pulse (pp74 and pp90, respectively), and no acoustic stimulus (i.e., only background noise was presented; st0). St100, st0, pp74, and pp90 trials were applied 10 times, st74 and st90 trials were applied five times, with trials randomly administered [average intertrial interval was 15 s (10–20 s)]. The background noise of each chamber was 70 dB. The data for startle amplitude were averaged across stimulus trials and the percent inhibition of the 110 dB startle by the 74 and 90 dB prepulse intensities was calculated for each animal to index PPI (Szumlinski et al., 2005a; Guo et al., 2016).

For studies of *Homer1a^+/+,+/-,-/-^* mice, behavioral testing occurred across days, with several tests conducted per day, spaced 4–6 h apart. The order of testing was the same for each cohort of animals: Day 1: PPI, reactivity to novel objects, Day 2: elevated plus-maze, and Porsolt swim test. Mice were then allowed 2–3 days recovery from the Porsolt swim test and then tested for their ability to habituate to repeated placement within a novel activity chamber (30 cm × 30 cm × 45 cm; 30 min sessions over 5 days). As the Porsolt swim test was predicted to reduce performance in a Morris water maze, a separate cohort of *Homer1a^+/+,+/-,-/-^* mice were trained (4, 2-min trials/day for 4 days) and then tested in the Morris water maze (2-min test) (see Lominac et al., 2005). Acoustic acuity was also assayed in a distinct cohort of male *Homer1a^+/+,+/-,-/-^* mice by randomly presenting acoustic stimuli ranging in intensity from 0 to 125 dB within the acoustic startle chambers during a 20-min session. Following this session, this cohort of animals were assayed for saccharin preference by presenting mice with two 50 ml sipper tubes in the home cage (water vs. 0.125% w/v saccharin) under continuous-access procedures over the course of 8 days. With the exception of this last cohort of mice, all behavioral experiments included approximately equal numbers of male and female subjects (minimum *n* = 6/sex/genotype) and sex was included as a between-subjects variable during data analysis by analyses of variance (ANOVA). However, with the exception of the novel object test, no sex differences in behavior were observed and thus, the data are presented collapsed across sex for the majority of the experiments.

For behavioral paradigms in which genotypic differences were observed, the experiment was replicated in *Homer1a^+/+^* and *Homer1a^-/-^* mice infused intra-NAC with an AAV carrying *Homer1a* cDNA (AAV-Homer1a) or a scrambled control (AAV-control; see section 2.4) to confirm an active role for this IEG isoform in regulating behavior. For these follow-up studies, behavioral testing commenced a minimum of 3 weeks following AAV infusion and mice were assayed for acoustic startle/PPI, followed by the Porsolt swim test. As behavior in the Morris Water Maze test relies heavily upon hippocampal, rather than accumbens, function, AAV-infused mice were not tested in this assay.

### 2.3 Cocaine-Induced Place-Conditioning

A separate cohort of male and female *Homer1a^+/+,+/-,-/-^* mice were used to index for genotypic differences in cocaine-conditioned reward using cocaine-induced place-conditioning. The procedures to elicit place-conditioning were identical to those described previously by our group (e.g., Ary et al., 2013), with separate groups of mice injected with intraperitoneal (i.p.) injections of 3, 10, or 30 mg/kg cocaine (National Institute on Drug Abuse, Bethesda, MD, United States). Animals were habituated to both compartments of the two-compartment apparatus for 15 min during a pre-conditioning testing (PreTest) and then were subjected to daily, 15-min, conditioning sessions, alternating between cocaine and saline, over the course of 8 days. The day following the last conditioning session, animals were allowed free-access to both compartments in a post-conditioning test. The behavior of the mice was recorded throughout the experiment using a digital video-tracking system and ANYMaze software (Stoelting, Co., Wood Dale, IL, United States). The difference in the time spent in the cocaine- versus saline-paired compartment on a post-conditioning test served to index cocaine reward/aversion (Conditioned Place-Preference Score or CPP Score). The data were analyzed using a Genotype (+/+, +/-, -/-) × Sex × Dose (3, 10, and 30 mg/kg cocaine) × Side (paired vs. unpaired) ANOVA, with repeated measures on the Side factor. The data for locomotor activity during the PreTest and cocaine-induced locomotion were published previously in Park et al. (2013). A follow-up study employed identical place-conditioning procedures to assay the effects of intra-NAC AAV-Homer1a infusion upon genotypic differences in novelty-induced locomotion during the PreTest, habituation of locomotor activity (changes in saline-induced locomotion observed during the four saline-conditioning sessions), sensitization of cocaine-induced locomotor hyperactivity (changes in locomotor activity observed during the four cocaine-conditioning sessions; 10 mg/kg) and cocaine-conditioned reward (CPP Score following conditioning).

### 2.4 Construction of AAV-Homer1a

The rat Homer1a coding sequence was amplified using whole-brain cDNA, and the PCR was product expressed as an N-terminal fusion protein with the hemagglutinin (HA)-tag in a rAAV backbone containing the 1.1 kb CMV enhancer/chicken-actin (CBA) promoter, 800 bp human interferon scaffold attachment region inserted 5_ of the promoter, the woodchuck post-transcriptional regulatory element (WPRE), and the bovine growth hormone polyA flanked by inverted terminal repeats (AAV-Homer1a, AAV-Homer1c). The same AAV-CBA-WPRE-bGH backbone encoding the enhanced green fluorescent protein (EGFP) was used as control (AAV-GFP). As described previously (Hauck et al., 2003), AAV pseudotyped vectors virions containing a 1:1 ratio of AAV1 and AAV2 capsid proteins with AAV2 intertrigeminal regions were generated. For this, human embryonic kidney 293 cells were transfected with the AAV *cis*plasmid, the AAV1 (pH21) and AAV2 (pRV1) helper plasmids (pF6), and the adenovirus helper plasmid by standard calcium phosphate transfection methods. Cells were harvested at 48 h after transfection, and the vector purified using heparin affinity columns as described previously (During et al., 2003). As also described previously (Clark et al., 1999), genomic titers were determined using the Prism 7700 sequence detector system (Applied Biosystems, Foster City, CA, United States) with primers designed to WPRE.

### 2.5 Surgical Procedures and AAV Infusion

The NAC is a limbic structure implicated in both the psychomotor-activing and rewarding/reinforcing properties of drugs of abuse (e.g., Morein-Zamir and Robbins, 2015; Scofield et al., 2016). Homer1a is induced within the NAC by various drugs of abuse (Szumlinski et al., 2008a; Marton et al., 2015) and *Homer1a^-/-^* mice exhibit impaired cocaine-induced neurochemical sensitization within this region (Park et al., 2013). Thus, under isoflurane anesthesia, we conducted craniotomies to implant bilateral guide cannulae (20-gauge; 10-mm long), 2 mm above the NAC of mice (see Lominac et al., 2005; Cozzoli et al., 2009; Ary et al., 2013), for the purposes of conducting *in vivo* microdialysis measurement of glutamate and dopamine (see section 2.6 below) and/or infusing AAVs carrying either cDNA for Homer1a (AAV-Homer1a) or a scrambled DNA sequence as a control (AAV-control) to determine an active role for NAC Homer1a expression in regulating neurochemistry and behavior (see Lominac et al., 2005; Cozzoli et al., 2009 for AAV details). A Kopf stereotaxic device held the animal’s head level and holes were drilled based on coordinates from bregma: (AP: +1.3 mm; ML: ±1.0 mm; DV: -2.3 mm) (Paxinos and Franklin, 2013). Guide cannulae were fixed to the skull with dental resin, surgical incisions were closed with a tissue adhesive.

For AAV-naïve mice undergoing microdialysis procedures, dummy cannulae (24 gauge; length equivalent to guide cannulae) was then placed inside the guide cannulae to prevent contamination or blockade and animals were allowed a minimum of 5 days recovery prior to neurochemical testing. Our AAVs are considered BSL1 and thus, standard protective personal equipment were employed during AAV infusion. For animals receiving AAV infusions, microinjectors (33-gauge, 12 mm long) where then lowered down the guide cannulae and the AAVs infused at a rate of 0.05 μl/min for 5 min (total volume/side = 0.25 μl). Microinjectors were left in place for an additional 5 min to allow for diffusion away from the microinjector tip prior to removal and occlusion of the guide cannulae. AAV-infused mice were allowed a minimum of 3 weeks recovery to achieve maximal neuronal transduction (Klugmann and Szumlinski, 2008). AAV transduction within the NAC was verified using standard immunohistochemical staining procedures for the hemagglutanin (HA) tag using a mouse anti-HA primary antibody (Covance, Princeton, NJ, United States), and straining visualized using a M.O.M. Detection Kit (Vector Laboratories, Burlingame, CA, United States), as conducted previously (Szumlinski et al., 2004, 2005b, 2017; Lominac et al., 2005; Cozzoli et al., 2009; Ary et al., 2013).

### 2.6 *In Vivo* Microdialysis

*Homer1a^-/-^* mice do not exhibit cocaine-induced dopamine or glutamate sensitization in the NAC (Park et al., 2013). Thus, a series of *in vivo* microdialysis experiments were conducted to relate this sensitization-resistant phenotype to biochemical indices of neuronal function within this region. For all in *vivo* microdialysis experiments, a microdialysis probe (24 gauge; 12 mm in length with ∼1.0 mm active membrane) was lowered unilaterally into one of the guide cannulae and perfused with artificial CSF (146 nM NaCl, 1.2 mM CaCl_2_, 2.7 mM KCl, 1.0 mM MgCl_2_, pH = 7.4) at a rate of 2 μl/min. Dialysate collection began after 3 h of probe equilibration and occurred in 20 min intervals into vials containing 10 μl of preservative [10% methanol (v/v), 15% acetonitrile (v/v), 150 mM NaPO_4_, 4.76 mM citric acid, 3 mM SDS, 50 mM EDTA, pH = 5.6]. Dialysate was stored at -80°C until analysis by HPLC (see section 2.6 below). Microdialysis probe localization within the NAC was verified using standard cresyl violet staining procedures (for AAV-naïve mice) or upon immunohistochemical localization of AAV transduction (see above), followed by examination of tissue under light microscope. In nearly all subjects, microdialysis probes were localized primarily to the shell subregion or to the interface between the shell and core subregions (see section 3).

#### 2.6.1 DHPG-Stimulated Glutamate Release

Homer proteins are critical regulators of mGlu1/5 function *in vitro* and *in vivo* (cf. Ango et al., 2000, 2001; Shiraishi-Yamaguchi and Furuichi, 2007; Worley et al., 2007; Szumlinski et al., 2008a; Fagni, 2012; Constantin, 2016). Thus, we compared the effects of *Homer1a* versus pan-*Homer1* deletion upon an *in vivo* index of mGlu1/5 function – agonist-stimulated glutamate release (Swanson et al., 2001; Szumlinski et al., 2004, 2017). For this, *Homer1a^+/+,-/-^* and pan-*Homer1^+/+,-/-^* mice were perfused within increasing concentrations of the mGlu1/5 agonist DHPG [(S)-3,5-dihydroxyphenylglycine; 0, 3, 30, and 300 μM; Tocris Cookson, Ballwin, MO, United States], via the microdialysis probe in 1-h intervals. For each mutant line, the data were analyzed using Genotype (+/+ vs. -/-) × DHPG ANOVA, with repeated measures on the DHPG factor (four levels).

#### 2.6.2 Depolarization-Induced Dopamine and Glutamate Release

To relate the distinct neurochemical phenotypes of *Homer1a* (Park et al., 2013) versus *Homer1* (Szumlinski et al., 2004, 2005a) mutants to the excitability of dopamine and glutamate terminals within the NAC, we compared depolarization-induced neurotransmitter release by locally infusing high K^+^ solutions (50 and 100 mM; Sigma-Aldrich, St. Louis, MO, United States) through the microdialysis probe in 1-h intervals (e.g., He and Shippenberg, 2000). To conserve animal numbers, this microdialysis experiment was conducted 3–4 days following the DHPG study described in Section 2.6.1 above, using the other side of the head. The data were analyzed separately for each mutant line using a Genotype × K^+^ ANOVA, with repeated measures on the K^+^ factor (three levels).

#### 2.6.3 Glutamate No Net-Flux Procedures

Pan-*Homer1* deletion reduces basal extracellular glutamate content within the NAC of mice (Szumlinski et al., 2005a) and prior conventional *in vivo* microdialysis studies suggested lower basal extracellular glutamate content within the NAC also of *Homer1a^-/-^* animals (Park et al., 2013). However, estimates of basal neurotransmitter levels assessed under conventional microdialysis procedures are subject to differences in probe recovery (Parsons and Justice, 1992). Thus, we quantified the effects of *Homer1a* deletion upon the basal extracellular glutamate content within the NAC using no net-flux procedures (Szumlinski et al., 2004, 2005a, 2017; Lominac et al., 2005; Ary et al., 2013). For this, increasing concentrations of glutamate (2.5, 5, and 10 μM) were infused through the probe in ascending order for 1 h each. Linear regression analyses were then conducted on the plot of the average net-flux of glutamate at each glutamate concentration versus the concentration of glutamate infused through the probe and the point of no net-flux (y = 0; estimate of basal extracellular levels of glutamate), as well as the slope of regression lines (estimate of glutamate clearance), were determined and analyzed using independent samples *t*-tests. A follow-up experiment determined how an intra-NAC AAV-Homer1a infusion altered the genotypic differences in our dependent measures in this assay. This follow-up study employed identical no net-flux procedures as those employed in the original genotypic comparison, with the exceptions that the *Homer1a^+/+,-/-^* mice were infused intra-NAC with either AAV-control or -Homer1a, a minimum of 3 weeks prior to probe insertion and the data were analyzed using an AAV (control vs. Homer1a) × Genotype (+/+ vs. -/-) ANOVA.

#### 2.6.4 Cocaine-Induced Sensitization of Dopamine and Glutamate within the NAC

To determine whether or not Homer1a within the NAC actively regulates the development of cocaine-induced dopamine and glutamate sensitization in this region, *Homer1a^+/+^* and *Homer1a^-/-^* mice were infused with AAV-control or -Homer1a and then conventional *in vivo* microdialysis procedures were conducted a minimum of 3 weeks later. Low fecundity in our Homer1a colony at the time of study forced us to conserve animal numbers and thus, microdialysis procedures were conducted in the same AAV-infused mice as those tested for cocaine-induced locomotor sensitization/place-preference (see section 2.3). In order to accommodate both paradigms, mice first underwent a microdialysis session to assay the effects of AAV infusion upon the neurochemical response to an acute injection of 30 mg/kg cocaine (i.p.). Following a 3-h equilibration period, baseline sampling was conducted over an hour and then mice were injected with cocaine and samples collected for 3 h thereafter. At the end of this first microdialysis session, the probe was removed and mice were allowed to recover for 2 days prior to the 10-day place-conditioning procedure (consisting of four alternating injections of saline and 30 mg/kg cocaine). One to 2 days following the post-conditioning test, a second microdialysis session was conducted using the opposite side of the head to assay for cocaine-induced neurochemical sensitization.

In contrast to our prior study (Park et al., 2013), the *Homer1a^+/+,-/-^* mice in this study were treated with a total of six injections of 30 mg/kg cocaine. Due to technical issues associated with subject attrition and sample loss due to technical difficulties with the HPLC, not all mice were tested for cocaine-induced neurochemical changes during both microdialysis sessions. Thus, in order to include all of the remaining the mice tested, Injection Number was treated as a between-subjects variable. As group differences were not observed for baseline glutamate levels on either injection, the data were expressed as a percent of baseline values for each group and to facilitate visualization of group differences in the magnitude of the cocaine-induced rise in dopamine and glutamate, the area under the curve (AUC) was employed in the statistical analyses of the results (Lominac et al., 2012; Shin et al., 2016). Thus, for both baseline neurotransmitter levels and the AUC for cocaine-induced changes in neurotransmitter levels, the data were analyzed using a Genotype × AAV × Injection Number between-subjects ANOVA.

### 2.7 High Pressure Liquid Chromatography

The high pressure liquid chromatography (HPLC) system and procedures for the electrochemical detection of glutamate and dopamine in the dialysate of mice, as well as the chromatographic analysis of the data, were identical to those described previously by our group (Lominac et al., 2012, 2014; Ary et al., 2013; Szumlinski et al., 2017). Each HPLC system consisted of a Coularray detector, a Model 542 autosampler and two Model 582 solvent delivery systems (ESA/Thermo-Fisher), with a detection limit of 0.01 fg/sample (20 μl/sample onto column). For analysis of dopamine, the MD-TM mobile phase was employed (ESA/Thermo-Fisher), and neurotransmitters in 30 μl from each 50 μl sample were separated using a MD-150 mm × 3.2 mm column (ESA/Thermo-Fisher). An ESA/Thermo-Fisher 5014B analytical cell was used for the detection of monoamines (oxidation and reduction electrode potentials of +220 and -150 mV, respectively). For glutamate, the mobile phase consisted of 3.5% acetonitrile (v/v), 22% methanol (v/v), 100 mM NaPO_4_, pH = 6.75. A reversed phase column (50 mm × 3.0 mm Capcell PAK; Shiseido, Tokyo, Japan) was used to separate the amino acids, and precolumn derivatization with *o*-phthaladehyde (2.7 mg/ml) of the 20 μl from each 50 μl sample was performed using an ESA Model 540 autosampler (ESA/Thermo-Fisher). Glutamate was detected using an electrochemical analytical cell with an oxidizing potential of +550 mV. Glutamate and dopamine content in each sample were analyzed by peak height and compared with an external standard curve for quantification (glutamate standards: 2.5, 5.0, 10 mM; dopamine standards: 1.25, 2.5, 5 nM).

### 2.8 General Statistical Approaches

As detailed above, the majority of data were analyzed using ANOVAs, with repeated measures as appropriate for the experimental design. Significant interactions were deconstructed along the relevant factors, and significant main effects were followed by least significant differences (LSDs) *post hoc* tests or *t*-tests, as appropriate for the number of comparisons. α = 0.05 for all analyses.

## 3 Results

### 3.1 Negative Affect

Exposure to a novel environment induces Homer1a expression within forebrain (Vazdarjanova et al., 2002) and pan-*Homer1* KO mice exhibit robust signs of hyper-emotionality across a variety of behavioral paradigms (Szumlinski et al., 2004, 2005a; Lominac et al., 2005). Thus, we first assayed *Homer1a^+/+^* and *Homer1a^-/-^* mice for behavioral signs of negative affect using a comparable behavioral test battery as that employed in our previous work. As detailed below, the results do not support a consistent role for Homer1a induction in the manifestation of anxiety- or depressive-like behaviors in mice.

#### 3.1.1 Elevated Plus Maze

**FIGURE 1:**
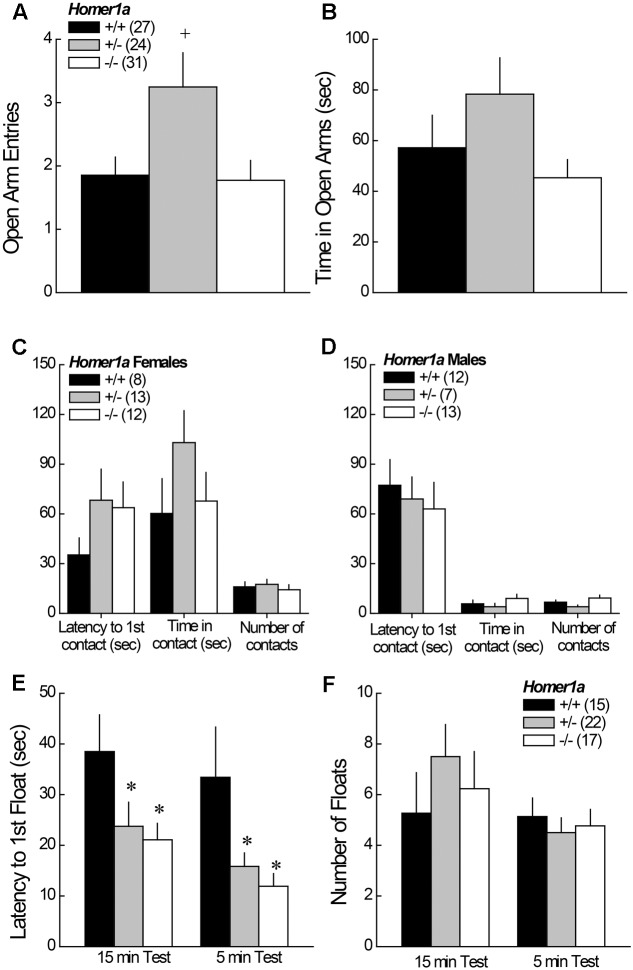
Anxiety-like behavior in *Homer1a^+/+^*, *Homer1a^+/-^*, and *Homer1a^-/-^* mice. When compared in an elevated plus maze test, *Homer1a^+/-^* mice exhibited relatively lower anxiety as indicated by greater **(A)** number of open arm entries and **(B)** time spent in the open arms. While no sex differences in anxiety-like behavior were observed on the elevated plus maze, females **(C)** exhibited greater anxiety than males **(D)**, in the novel object test, but no genotypic differences were observed. No sex differences were apparent in the Porsolt Swim test, however, Homer1a deletion increased **(E)** the latency to first float, without influencing the total amount of floating **(F)**. The data represent the mean ± SEM of the number of mice indicated in parentheses. ^∗^*p* < 0.05 vs. +/+; +*p* < 0.05 vs. +/+ and *–*/*–* (LSD *post hoc* tests).

When tested in the elevated plus maze test, no main effects or interactions with sex were detected (*p* > 0.05 for all variables); thus, the data for male and female mice were collapsed for data presentation. *Homer1a^+/-^* mice exhibited a significant increase in open arm entries, relative to both *Homer1a^+/+^* and *Homer1a^-/-^* mice (**Figure 1A**) [Genotype effect: *F*(1,79) = 4.50, *p* = 0.01; LSD *post hoc* tests]. A similar pattern of genotypic differences was observed for the time spent in the open arms, but the result was shy of statistical significance (**Figure 1B**; Genotype effect: *p* = 0.1).

#### 3.1.2 Novel Object Test

In the novel object test assay, females exhibited lower behavioral indices of anxiety than males, as indicated by a significant increase in both the time in object contact [Sex effect: *F*(1,59) = 18.91, *p* < 0.0001], and number of object contacts [Sex effect: *F*(1,59) = 36.50, *p* < 0.0001]. No sex difference was observed for the latency to first make contact with the novel object (*p* = 0.31) and, as illustrated in **Figure 1C** (females) and **Figure 1D** (males), genotypic differences in exploratory behavior were not observed for either sex (*p* > 0.20 for all variables).

#### 3.1.3 Porsolt Swim Test

No sex differences were observed for any of the variables measured during Porsolt Swim testing (Sex effects and interactions, *p*’s > 0.05). Thus, the data were collapsed across sex for presentation. *Homer1a* deletion reduced the latency to first float during both the 15-min exposure session, as well as during the 5-min re-exposure session conducted 24 h later (**Figure 1E**), with both *Homer1a^+/-^* and *Homer1a^-/-^* mice exhibiting a shorter latency to float, compared to controls [Test effect: *p* = 0.08; Genotype effect: *F*(2,51) = 6.5, *p* = 0.003; Genotype × Test: *p* = 0.93]. However, no genotypic differences were observed regarding the total floating during either session (**Figure 1F**) [Test effect: *F*(1,51) = 3.93, *p* = 0.05; Genotype effect and Genotype × Test: *p* > 0.31].

#### 3.1.4 Saccharin Preference

Although the results of the Porsolt swim test suggested greater depressive-like behavior in *Homer1a^-/-^* mice, we failed to detect genotypic differences in the initial or average preference for a palatable saccharin solution over water (for both variables, Genotype effects, *p* > 0.25), with the average preference exhibited by *Homer1a^+/+^*, *Homer1a^+/-^*, and *Homer1a^-/-^* mice being 43.1 ± 3.80, 50.90 ± 2.49, and 45.11 ± 4.48%, respectively.

#### 3.1.5 Habituation to a Novel Environment

As the above data argue an inconsistent role for *Homer1a* in mediating the behavioral response to an acute mild stressor, we next determined whether *Homer1a* deletion might impair the ability to habituate to the repeated presentation of a mild stressor using a simple locomotor habituation paradigm. No significant main effects of, or interactions with, the Sex factor were observed (*p*’s > 0.15). While there were trends for reduced overall locomotor activity in *Homer1a^+/-^* and *Homer1a^-/-^* mice, this genotypic difference was not significant nor were there genotypic differences in the extent to which animals habituated with repeated testing (data not shown) [Session effect: *F*(4,208) = 23.41, *p* < 0.0001; Genotype effect and Genotype × Session, *p* > 0.05]. Further, no genotypic differences were observed for the change in the latency to enter the center zone (data not shown) [Session effect: *p* = 0.09; Genotype × Session, *p* > 0.05] or in the number of entries into the center zone of the activity monitors with repeated testing (data not shown) [Session effect: *F*(4,52) = 5.33, *p* < 0.0001; Genotype × Session: *p* > 0.95].

### 3.2 Sensorimotor Processing

Pan-*Homer1* knock-out mice exhibit marked deficits in sensorimotor-gating (Szumlinski et al., 2004, 2005a; Lominac et al., 2005). Thus, we determined the effects of *Homer1a* deletion upon acoustic startle and pre-pulse inhibition of acoustic startle (PPI). Again, we failed to detect a sex difference in behavior (for all variables, no Sex effect or interactions, *p* > 0.30), thus, the data were collapsed across sex for presentation.

#### 3.2.1 Acoustic Startle during PPI Testing

**FIGURE 2:**
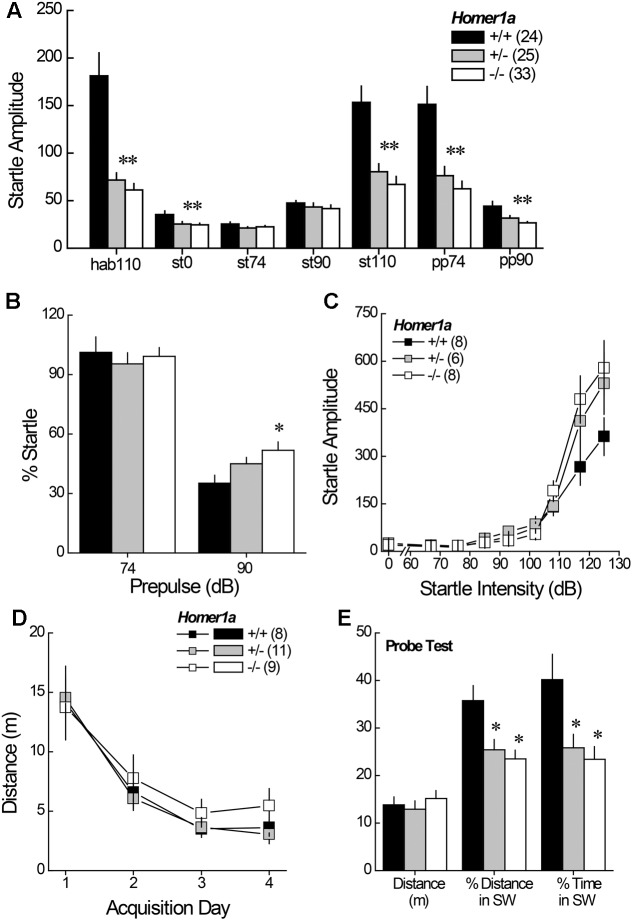
Sensorimotor processing and spatial memory in *Homer1a^+/+^*, *Homer1a^+/-^*, and *Homer1a^-/-^* mice. **(A)** Both *Homer1a^+/-^* and *Homer1a^-/-^* mice exhibited reduced startle amplitude during testing PPI testing. **(B)** Only *Homer1a^-/-^* mice exhibited impaired PPI at the 90 dB pre-pulse. **(C)** No overt genotypic differences were apparent in acoustic acuity across a range of auditory stimuli. **(D)** No overt genotypic differences were observed over 4 days of training in the acquisition of a Morris water maze task. **(E)** However, *Homer1a^+/-^* and *Homer1a^-/-^* mice were impaired in their ability to recall the platform location, 24 h later. The data represent the mean ± SEM of the number of mice indicated in parentheses. ^∗^*p* < 0.05 vs. +/+ (LSD *post hoc* tests).

Relative to *Homer1a^+/+^* littermates, both *Homer1a^+/-^* and *Homer1a^-/-^* mice exhibited reduced startle responsiveness to the various acoustic stimuli (**Figure 2A**) [Genotype effect: *F*(2,79) = 21.95, *p* < 0.0001; Tone effect: *F*(6,474) = 84.60, *p* < 0.0001; Genotype × Tone: *F*(12,474) = 14.4, *p* < 0.0001]. Relative to controls, lower startle-responding to the 110 dB tone was observed in both *Homer1a^+/-^* and *Homer1a^-/-^* mice during the habituation phase (hab110), when it was presented alone (st110) or when it was preceded by either the 74 or the 90 dB pre-pulse (*p*’s < 0.007; LSD *post hoc* tests). Moreover, both *Homer1a^+/-^* and *Homer1a^-/-^* mice exhibited lower spontaneous activity than *Homer1a^+/+^* animals in the absence of any acoustic stimuli (st0; *p* = 0.006).

#### 3.2.2 PPI

While the 74 dB pre-pulse failed to inhibit acoustic startle in any genotype (*p* > 0.25), a genotypic difference in PPI was observed in the presence of the 90 dB pre-pulse (**Figure 2B**) [Genotype effect: *F*(2,79) = 4.59, *p* = 0.01]. Further, a significant impairment in PPI was observed at the 90 dB prepulse in *Homer1a^-/-^* mice, relative to controls (*p* = 0.003), with a modest impairment observed in *Homer1a^+/-^* mice (*p* = 0.09).

#### 3.2.3 Acoustic Acuity

Although the tone-dependency of the startle response exhibited by *Homer1a^+/-^* and *Homer1a^-/-^* mice during PPI testing (**Figure 2A**) argued that *Homer1a* deletion does not impair the ability to discriminate between certain acoustic stimuli, the unexpected blunting of the startle-responsiveness of the mutant mice prompted a test for a primary hearing deficit. For this, male *Homer1a^+/+^*, *Homer1a^+/-^*, and *Homer1a^-/-^* mice were randomly presented with acoustic stimuli ranging in intensity from 0 to 125 dB. In this cohort, startle amplitude was also intensity-dependent (**Figure 2C**) [Tone effect: *F*(8,152) = 82.29, *p* < 0.0001]; however, although the results of the statistical analyses indicated a significant interaction between genotype and tone [*F*(8,152) = 2.36, *p* = 0.004], *post hoc* comparisons at each dB level failed to reveal genotypic differences in startle responsiveness.

### 3.3 Spatial Learning and Memory

Pan-*Homer1* deletion markedly impairs learning and memory on several assays (Lominac et al., 2005; Szumlinski et al., 2005a; Gerstein et al., 2012) and intracranial manipulations of Homer1a expression affect spatial memory (e.g., Klugmann et al., 2005; Celikel et al., 2007; Gerstein et al., 2012, p. 213). Thus, we trained male mice to locate a hidden platform in a Morris water maze task and then assessed for long-term spatial memory in a probe trial, conducted 24 h following the 4th acquisition session. Surprisingly, genotypic differences in behavior were not detected during the acquisition phase of testing, either across trials within days, or across days of training (**Figure 2D**) [distance to locate platform, Trial effect: *F*(3,225) = 5.18, *p* = 0.003; Day effect: *F*(3,225) = 33.50, *p* < 0.0001; no main effect of, or interactions with the Genotype factor; data not shown for time taken to locate platform, Trial effect: *F*(3,225) = 7.87, *p* < 0.0001; Day effect: *F*(3,225) = 34.46, *p* < 0.0001; no main effect of, or interaction with, the Genotype factor]. In contrast to the data for maze acquisition, marked genotypic differences were observed regarding recall of the platform location, when tested 24 h later (**Figure 2E**), with both *Homer1a^+/-^* and *Homer1a^-/-^* mice exhibiting impaired recall, as indicated by lower percent total distance and percent time spent in the SW quadrant, which formerly contained the hidden platform [for % Distance: Genotype effect: *F*(2,27) = 6.79, *p* = 0.004; for % Time: Genotype effect: *F*(2,27) = 5.82, *p* = 0.008; *post hoc* tests]. No genotypic difference in the distance swam (**Figure 2E**; *p* = 0.65) argues that the recall impairment exhibited by Homer1a mutants was unrelated to swimming ability.

### 3.4 Cocaine-Conditioned Place-Preference

**FIGURE 3:**
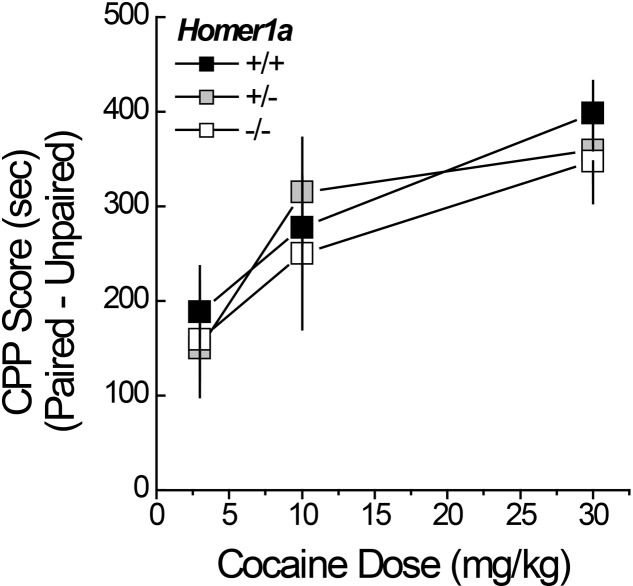
Cocaine-induced place-conditioning in *Homer1a^+/+^*, *Homer1a^+/-^*, and *Homer1a^-/-^* mice. When tested in a cocaine-free state, genotypic differences were not observed in the dose-response function for cocaine-induced place-preference exhibited by male and female mice. Occupancy difference = time spent on the cocaine-paired minus -unpaired side in seconds). The data represent the mean ± SEM of the number of mice indicated in parentheses.

Pan-*Homer1* deletion increases sensitivity to the conditioned rewarding properties of cocaine (Szumlinski et al., 2004; Lominac et al., 2005). Thus, we characterized the dose-response function for cocaine-induced place-conditioning in male and female *Homer1a^+/+,+/-,-/-^* mice. No sex differences were observed for cocaine-induced place-conditioning [Side effect: *F*(1,167) = 223.67, *p* < 0.000; Dose × Side: *F*(2,167) = 11.36, *p* < 0.0001; Sex effect and interactions, *p*’s > 0.10] and so, the data were collapsed across sex for presentation. Despite the large sample sizes employed in this experiment, we did not detect an effect of *Homer1a* deletion upon the dose-response function for cocaine-induced place-preference (**Figure 3**; no Genotype effect or Genotype × Side interactions, *p*’s > 0.35). These data argue little role for Homer1a induction in either the associative learning/memory or incentive motivational processes involved in the development and expression of cocaine-conditioned reward.

### 3.5 DHPG-Induced Glutamate Release

**FIGURE 4:**
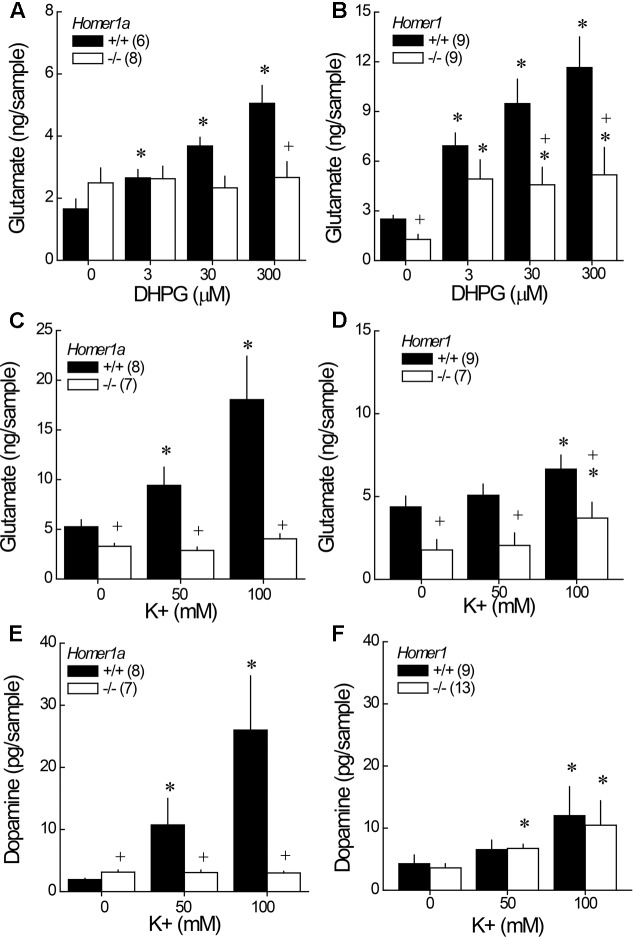
Comparison of the effects of *Homer1a* versus pan-*Homer1* deletion upon the regulation of NAC glutamate and dopamine. Intra-NAC perfusion with increasing concentrations of the mGlu1/5 agonist DHPG failed to increase extracellular glutamate in *Homer1a^-/-^* mice **(A)**, while *Homer1^-^*^/^*^-^* mice exhibited a moderate, but significant, DHPG-stimulated rise in NAC glutamate **(B)**. Intra-NAC perfusion with high K^+^ solutions elevated NAC glutamate levels and this effect was markedly blunted in *Homer1a^-/-^* mice **(C)**, but not in *Homer1^-/-^* mice, although *Homer1^-/-^* mice exhibited lower glutamate levels, overall **(D)**. *Homer1a^-/-^* mice failed to exhibit K^+^-stimulated dopamine release **(E)**, while dopamine release was unaffected in *Homer1^-/-^* mice **(F)**. The data represent the mean ± SEM of the number of mice indicated in parentheses. ^∗^*p* < 0.05 vs. 0 μM; +*p* < 0.05 vs. *Homer1a^+/+^* or *Homer1^+/+^*.

Although it appeared as if *Homer1a* deletion elevated baseline glutamate in the study of DHPG-induced glutamate release (**Figure 4A**; DHPG = 0 μM), the genotypic difference was not significant (*t*-test: *p* = 0.19). However, *Homer1a* deletion prevented the capacity of DHPG to stimulate glutamate release within NAC (**Figure 4A**) [Genotype × Dose: *F*(3,36) = 26.02, *p* < 0.0001]. DHPG dose-dependently elevated glutamate in *Homer1a^+/+^* mice [DHPG effect: *F*(3,15) = 68.31, *p* < 0.0001], while no DHPG-induced rise was apparent in *Homer1a^-/-^* mice (DHGP effect: *p* = 0.70). In contrast to *Homer1a* deletion, a comparison of baseline glutamate indicated lower glutamate levels in *Homer1^-/-^* versus *Homer1^+/+^* mice [*t*(16) = 2.36, *p* = 0.02]. Further, pan-*Homer1* deletion blunted, but did not block, the capacity of DHPG to increase NAC glutamate (**Figure 4B**) [Genotype × DHPG: *F*(3,51) = 4.65, *p* = 0.006], as evidenced by a significant DHPG-induced rise in glutamate in both genotypes [DHPG effect, for *Homer1^+/+^*: *F*(3,24) = 15.00, *p* < 0.0001; for *Homer1^-^*^/^*^-^*: *F*(3,27) = 4.37, *p* < 0.01]. These data provide evidence for distinct effects of *Homer1a* and pan-*Homer1* deletion upon mGlu1/5-dependent regulation of extracellular glutamate *in vivo*.

### 3.6 Depolarization-Induced NAC Glutamate and Dopamine Release

In this study, both *Homer1a^-/-^* (**Figure 4C**) and pan-*Homer1^-/-^* (**Figure 4D**) mice exhibited significantly lower baseline glutamate levels than their wild-type controls [for *Homer1a*: *t*(13) = 2.39, *p* = 0.03; for pan-*Homer1*: *t*(14) = 3.02, *p* = 0.009]. In contrast, *Homer1a^-/-^* KO mice exhibited a modest, but statistically significant elevation, in basal dopamine levels (**Figure 4E**) [*t*(13) = 2.05, *p* = 0.045], whereas differences in basal dopamine were not apparent between *Homer1^+/+^* and *Homer1^-/-^* mice (**Figure 4F**; *t*-test, *p* > 0.35).

Despite employing the same perfusate through the course of study, the effects of depolarization were more pronounced in the *Homer1a* versus the *Homer1* study, which might reflect differences in the genetic background of the two mutant lines. Nevertheless, a marked genotypic difference in K^+^-stimulated glutamate release was apparent in the study of *Homer1a* mice [Genotype × K^+^: *F*(2,26) = 7.93, *p* = 0.002]; while, K^+^ stimulated a rise in glutamate in both genotypes (**Figure 4C**) [for *Homer1a^+/+^*: *F*(2,14) = 10.70, *p* = 0.002; for *Homer1a^-/-^*: *F*(2,12) = 6.01, *p* = 0.02], the magnitude of the rise observed in *Homer1a^-/-^* animals was considerably less than that observed in their controls. Although, *Homer1^-/-^* mice exhibited lower glutamate levels than *Homer1^+/+^* animals, irrespective of the concentration of K^+^ perfused (**Figure 4D**) [Genotype effect: *F*(1,14) = 9.67, *p* = 0.008], *Homer1* deletion did not alter the capacity of K^+^ to stimulate glutamate release within the NAC [K^+^ effect: *F*(2,28) = 13.08, *p* < 0.0001; Genotype × K^+^: *p* = 0.11].

In the case of dopamine, *Homer1a* deletion prevented K^+^-stimulated dopamine release within the NAC (**Figure 4E**) [Genotype × K^+^: *F*(2,38) = 4.57, *p* = 0.02; one-way ANOVAs: for *Homer1a^+/+^*, *F*(2,26) = 9.32, *p* = 0.001; for *Homer1a^-/-^*: *p* = 0.78]. In contrast, pan-*Homer1* deletion had no impact upon K^+^-stimulated dopamine release in this region [K^+^ effect: *F*(2,40) = 6.86, *p* = 0.003; Genotype effect and interaction, *p*’s > 0.70]. These data provide novel evidence that the selective deletion of *Homer1a*, but not the entire *Homer1* gene, reduces the excitability of both dopamine and glutamate terminals within the NAC.

### 3.7 Glutamate No Net-Flux

**FIGURE 5:**
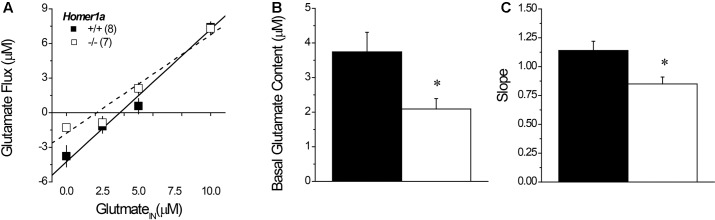
Basal extracellular glutamate content and release/clearance in *Homer1a^+/+^* and *Homer1a^-/-^* mice. Summary of the results of the linear regression analyses of the data from the no net-flux study of extracellular glutamate **(A)**, highlighting **(B)** the lower x-intercept (an estimate of basal glutamate content) and **(C)** reduced slope (an index of reuptake/release) in *Homer1a^-/-^* versus *Homer1a^+/+^* animals. The data represent the mean ± SEM of the number of mice indicated in parentheses in **(A)**. ^∗^*p* < 0.05 vs. +/+.

Linear regression analyses of the plots of the no net-flux study (**Figure 5A**) confirmed lower NAC extracellular glutamate content in *Homer1a^-/-^* versus *Homer1a^+/+^* mice (**Figure 5B**) [*t*(13) = 5.85, *p* = 0.03]. Linear analysis also indicated a significant reduction in the *in vivo* recovery of glutamate in *Homer1a^-/-^* mice relative to *Homer1a^+/+^* controls, as indicated by differences in the slopes of the linear regressions (**Figure 5C**) [*t*(13) = 5.51, *p* = 0.04].

### 3.8 AAV-Homer1a Effects upon Genotypic Differences in Behavior in Drug-Naïve Animals

To examine an active role for nucleus accumbens Homer1a in the behavioral phenotype of *Homer1a^-/-^* mice, AAV constructs carrying either Homer1a cDNA (AAV-Homer1a) or a scrambled DNA control were infused into the nucleus accumbens of *Homer1a^+/+^* and *Homer1a^-/-^* mice.

#### 3.8.1 Acoustic Startle and PPI

**FIGURE 6:**
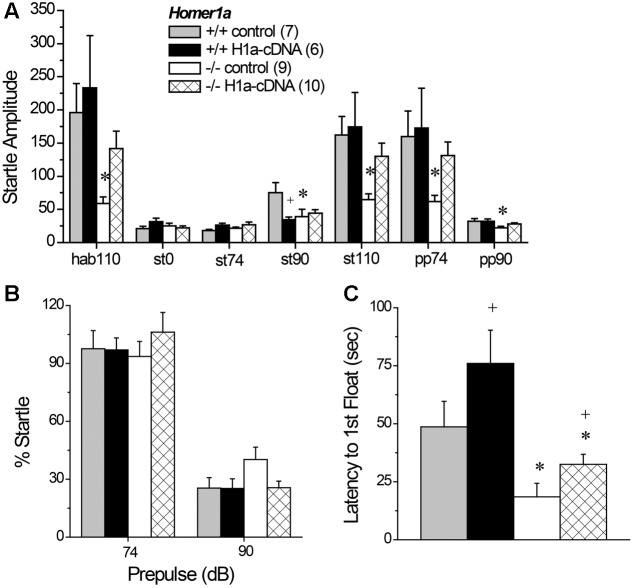
The effects of AAV-mediated restoration of Homer1a expression in the NAC of *Homer1a^-/-^* mice upon sensorimotor function and depressive-like behavior. **(A)** Genotypic differences in acoustic startle were apparent only between *Homer1a^+/+^* and *Homer1a^-/-^* mice infused with AAV-control (con). **(B)** Group differences were not observed for PPI. **(C)** *Homer1a^-/-^* mice exhibited a shorter latency to float in a 15-min Porsolt swim test and AAV-Homer1a (H1a) increased the latency to float in both genotypes. The data represent the mean ± SEM of the number of mice indicated in parentheses. ^∗^*p* < 0.05 vs. respective +/+; +*p* < 0.05 vs. respective AAV control.

We first employed Tukey tests for planned comparisons to compare between *Homer1a^+/+^* and *Homer1a^-/-^* mice infused with the control AVV to confirm replication of our prior genotypic difference in startle amplitude. Indeed, we replicated lower startle responsiveness in *Homer1a^-^*^/^*^-^* mice, specifically during habituation to the 110 dB stimulus (*p* = 0.04), in response to the 90 dB tone (*p* = 0.02), the 110 dB tone (*p* = 0.04) and in response to the 110 dB tone when preceded by the 74 or the 90 dB prepulse (both *p*’s = 0.05). We did not observe a genotypic difference between AAV-controls at the 0 or 74 dB stimuli or when the 110 dB stimulation was preceded by the 90 dB prepulse (*p*’s > 0.90). Planned comparisons were then conducted between AAV-control- and AAV-Homer1a-infused animals within each genotype. Despite what appeared to be a relatively selective effect of *Homer1a* over-expression upon the startle responsiveness of *Homer1a^-/-^* mice (**Figure 6A**), this statistical approach yielded no AAV-Homer1a effects in either genotype at any of the stimulus intensities (*p*’s > 0.20), with the exception of a significant reduction in the response exhibited by *Homer1a^+/+^* mice to the 90 dB tone (*p* = 0.02; st90 in **Figure 6A**). However, a comparison of the startle amplitude exhibited between *Homer1a^+/+^* and *Homer1a^-^*^/^*^-^* mice infused intra-NAC with AAV-Homer1a indicated no significant genotypic differences at any of the stimulus intensities (*p*’s > 0.40), arguing that AAV-Homer1a infusion into the NAC was sufficient to reverse the blunted sensorimotor-processing induced by *Homer1a* deletion.

A comparable analysis of PPI (**Figure 6B**) detected no group differences at the 74 dB prepulse (for all comparisons, *p*’s > 0.70) or at the 110 dB prepulse (for all comparisons, *p*’s > 0.10). Thus, we failed to replicate the PPI deficit observed in AAV-naïve mice (**Figure 2B**), nor did we find evidence to support an active role for NAC Homer1a in regulating the magnitude of PPI.

#### 3.8.2 Porsolt Swim Test

Replicating the data in **Figure 1E**, *Homer1a* deletion reduced the latency to first exhibit floating [Genotype effect: *F*(1,29) = 15.93, *p* < 0.0001]; however, intra-NAC AAV-Homer1a infusion increased the latency first float, irrespective of genotype (**Figure 6C**) [AAV effect; *F*(1,29) = 5.00, *p* = 0.03; interaction: *p* = 0.48], with AAV-Homer1a partially reversing the behavior of *Homer1^-/-^* mice. In contrast, we did not replicate the genotypic difference in total floating nor did we observed any AAV effect upon total floating in this experiment (data not shown; Genotype × AAV ANOVA, all *p*’s > 0.35). Further, AAV infusion did not impact the distance traveled during swim testing (data now shown; Genotype × AAV ANOVA, all *p*’s > 0.55).

### 3.9 AAV-Homer1a Effects upon Genotypic Differences in Cocaine-Induced Behavior

Although we failed to detect an effect of *Homer1a* deletion upon cocaine-conditioned reward (**Figure 3**), *Homer1a* deletion prevents the development of cocaine-induced behavioral sensitization (Park et al., 2013) and the effect of Homer1a over-expression within the NAC is not known. Thus, we employed our cocaine-induced place-conditioning procedures to determine the effects of intra-NAC AAV-Homer1a infusion upon spontaneous and cocaine-induced changes in locomotor activity, as well as place-conditioning, in *Homer1a^+/+^* and *Homer1a^-/-^* mice.

#### 3.9.1 Locomotor Reactivity to Novelty and Habituation

**FIGURE 7:**
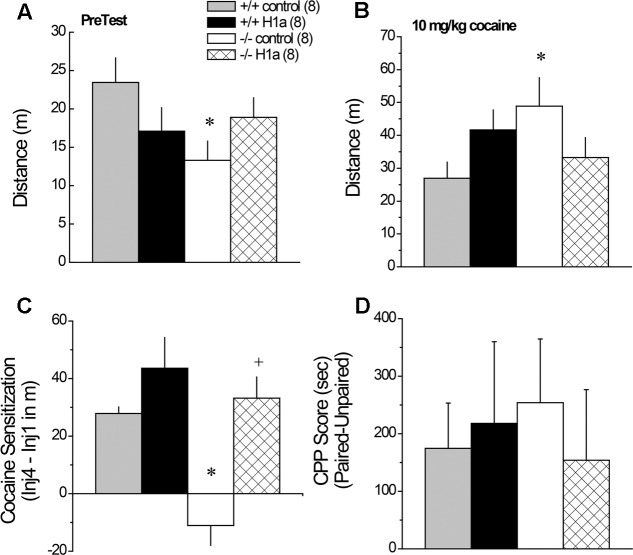
The effects of AAV-mediated restoration of Homer1a expression in the NAC of *Homer1a^-/-^* mice upon behavior in a cocaine-conditioned place-preference assay. Genotypic differences were observed only in mice infused with AAV-control for both the total distance traveled during the 15-min PreTest **(A)** and the distance traveled in response to an acute injection of 10 mg/kg cocaine **(B)**. **(C)** *Homer1a^-/-^* controls failed to exhibit cocaine-induced sensitization and this phenotype was reversed by AAV-Homer1a infusion. **(D)** Group differences were not observed for the magnitude of a conditioned place-preference elicited by four pairings of 10 mg/kg cocaine. The data represent the mean ± SEM of the number of mice indicated in parentheses. ^∗^*p* < 0.05 vs. respective +/+; +*p* < 0.05 vs. respective AAV control.

A significant Genotype by AAV interaction was observed for the locomotor hyperactivity expressed when mice were allowed to explore the place-conditioning apparatus on the PreTest (**Figure 7A**) [Genotype × AAV: *F*(1,31) = 4.45, *p* = 0.04]. This interaction reflected genotypic differences between *Homer1a^+/+^* and *Homer1a^-/-^* mice infused with AAV-control (*p* = 0.02), which was not apparent in mice infused with AAV-Homer1a (*p* = 0.70). When compared to their respective AAV-control, there was no detectable effect of AAV-Homer1a upon the novelty-induced locomotion of either *Homer1a^+/+^* (*p* = 0.12) or *Homer1a^-/-^* mice (*p* = 0.18). No group differences were observed with respect to either the locomotor response to an acute saline injection (Genotype × AAV ANOVA, all *p*’s > 0.65) or the change in saline-induced locomotion across the four saline-conditioning sessions (data not shown) [Saline Injection effect: *F*(3,84) = 4.69, *p* = 0.04; all other *p*’s > 0.07].

#### 3.9.2 Cocaine-Induced Locomotion and Sensitization

Interestingly, the group differences in the acute locomotor response to an acute injection of 30 mg/kg cocaine was inverse of that observed for novelty-induced locomotion (**Figure 7A** vs. **7B**) [Genotype × AAV: *F*(1,28) = 5.30, *p* = 0.03], with significant genotypic differences observed between *Homer1a^+/+^* and *Homer1a^-/-^* mice infused with AAV-control (*p* = 0.03), but not mice infused with AAV-Homer1a (*p* = 0.38). Further, we observed no effect of AAV-Homer1a upon acute cocaine-induced locomotion in either *Homer1a^+/+^* (*p* = 0.13) or *Homer1a^-/-^* mice (*p* = 0.11).

In contrast to the locomotor response to acute cocaine, AAV-Homer1a infusion completely reversed the effects of *Homer1a* deletion upon the development of cocaine-induced locomotor sensitization [Genotype × AAV × Cocaine Injection: *F*(3,84) = 4.94, *p* = 0.003]. Consistent with prior results for AAV-naïve mice (Park et al., 2013), *Homer1a^+/+^* mice infused with AAV-control exhibited robust sensitization across the four cocaine injections [*F*(3,21) = 15.23, *p* < 0.0001], as did *Homer1a^+/+^* mice infused with AAV-Homer1a [*F*(3,21) = 12.37, *p* < 0.0001]. While cocaine-induced locomotion did vary as a function of repeated cocaine treatment in *Homer1a^-/-^* infused with AAV-control [*F*(3,21) = 4.33, *p* = 0.02], this reflected a decline, rather than an increase, in activity with repeated cocaine treatment. In contrast, *Homer1a^-/-^* mice infused with AAV-Homer1a exhibited an injection-dependent increase in cocaine-induced locomotion [*F*(3,21) = 8.36, *p* = 0.001], indicative of restored sensitization.

To facilitate visualization of the group differences in cocaine-induced sensitization, the data are presented as difference in locomotor activity from injection 1 to 4 of repeated treatment (**Figure 7C**). Analysis of these data further indicates a reversal of the cocaine-sensitization phenotype of *Homer1a^-/-^* mice [Genotype × AAV: *F*(1,31) = 4.21, *p* = 0.05], with *Homer1a^-/-^* AAV-control exhibiting a locomotor phenotype distinct from the other groups tested (*p*’s ≤ 0.001). Although AAV-Homer1a infusion increased the magnitude of the cocaine-sensitized response in both genotypes, the AAV effect was significant only in *Homer1a^-/-^* mice (*Homer1a^+/+^*: *p* > 0.20; *Homer1a^-/-^*: *p* < 0.0001).

#### 3.9.3 Cocaine-Conditioned Place-Preference

**FIGURE 8:**
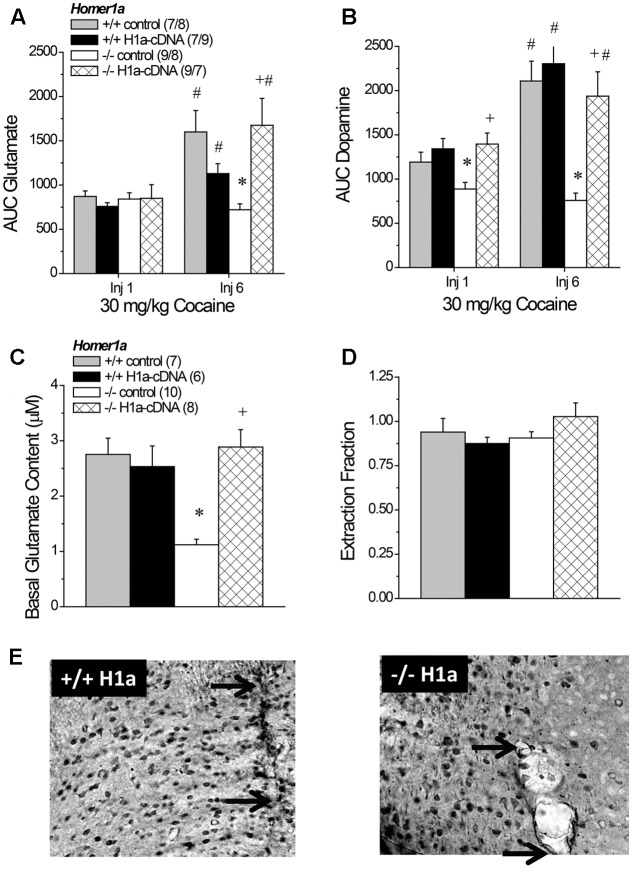
The effects of AAV-mediated restoration of Homer1a expression in *Homer1a^-/-^* mice upon basal and cocaine-stimulated neurotransmission. **(A)** Under conventional *in vivo* microdialysis procedures, repeated treatment with 30 mg/kg cocaine sensitized cocaine-induced glutamate release in both *Homer1a^+/+^* infused intra-NAC with AAV, but only in *Homer1a^-/-^* mice infused with AAV-Homer1a (H1a). **(B)** Intra-NAC AAV-Homer1a infusion increased the magnitude of the cocaine-elicited rise in dopamine in *Homer1a^-/-^* mice and facilitated the expression of dopamine sensitization with repeated cocaine treatment. The sample sizes in **(B)** are presented in **(A)**. When determined using glutamate no net-flux procedures, intra-NAC AAV-Homer1a reversed the low basal glutamate phenotype of *Homer1a^-/-^* mice **(C)**, but did not alter the slope of linear regressions in either genotype **(D)**. The samples sizes in **(D)** are presented in **(C)**. The data in **(A–D)** represent the mean ± SEM of the number of mice indicated in parentheses. ^∗^*p* < 0.05 vs. respective +/+; +*p* < 0.05 vs. respective AAV control; #*p* < 0.05 vs. Injection 1 (sensitization). **(E)** Representative images of HA-staining within the NAC of *Homer1a^+/+^* (left) and *Homer1a^-/-^* mice (right) infused with AAV-Homer1a (20X magnification), illustrating comparable neuronal transduction around the site of microinjection/microdialysis probe insertion. Arrows in **(E)** represent the extent of the microdialysis probe membranes.

Relative to their sensitized locomotor response to cocaine (**Figure 7C**), the magnitude of a cocaine-conditioned place-preference was highly variable (**Figure 7D**) and we detected no effect of genotype or AAV treatment upon the CPP Score exhibited by the mice when tested for compartment preference in a cocaine-free state (**Figure 8D**; Genotype × AAV ANOVA on CPP Score, *p*’s > 0.45).

### 3.10 AAV-Homer1a Effects on Basal and Cocaine-Stimulated Neurotransmission

#### 3.10.1 Cocaine-Induced Glutamate Sensitization

An analysis of the average baseline glutamate levels obtained using conventional *in vivo* microdialysis procedures failed to detect any group differences (data not shown; Genotype × AAV × Injection Number ANOVA, *p*’s > 0.15). As expected, the capacity of cocaine to increase NAC glutamate was injection-dependent [Injection Number effect: *F*(1,67) = 16.04, *p* < 0.0001]. However, the magnitude of the cocaine-sensitized glutamate response varied as a function of both genotype and AAV treatment [Genotype × AAV × Injection Number: *F*(1,67) = 8.36, *p* = 0.005]. Neither genotype nor AAV infusion influenced the glutamate response to acute cocaine (Genotype × AAV ANOVA: all *p*’s > 0.45), while AAV-Homer1a infusion augmented the capacity of the 6th cocaine injection to increase glutamate response only in *Homer1a^-/-^* mice [Genotype × AAV: *F*(1,32) = -10.58, *p* = 0.003]. Indeed, all groups but the *Homer1a^-/-^* controls exhibited greater cocaine-induced glutamate release on the 6th versus the 1st cocaine injection (for *Homer1a^-/-^* AAV-control: *p* = 0.23; for all other groups, *p*’s < 0.04).

#### 3.10.2 Cocaine-Induced Dopamine Sensitization

As observed for glutamate, there were no group differences in baseline levels of dopamine (data not shown; Genotype × AAV × Injection Number ANOVA, all *p*’s > 0.06) and repeated cocaine treatment elicited a robust increase in dopamine that varied as a function of Genotype [Injection Number effect: *F*(1,67) = 21.76, *p* < 0.0001; Genotype × Injection Number: *F*(1,67) = 9.00, *p* = 0.004], but not AAV infusion (AAV × Injection Number: *p* = 0.15). Although the magnitude of the dopamine response to cocaine was greater following repeated treatment, the effects of AAV infusion upon the genotypic differences in dopamine responsiveness were similar across the microdialysis sessions (**Figure 8B**) [Genotype × AAV: *F*(1,67) = 7.46, *p* = 0.008; three-way interaction: *p* = 0.21], with *Homer1a^-/-^* controls exhibiting a blunted dopamine response to cocaine, relative to their *Homer1^+/+^* counterparts (p) and AAV-Homer1a augmenting the cocaine-induced rise in dopamine only in Homer1a*^-^*^/^*^-^* animals (for *Homer1a^+/+^*: *p* = 0.57; *Homer1a^-/-^*: *p* < 0.0001).

#### 3.10.3 No Net-Flux Glutamate

A planned comparison of the genotypic difference in the x-intercept for mice infused with AAV-control replicated lower basal glutamate content in *Homer1a^-/-^* mice (*p* < 0.0001). We also detected a significant Genotype × AAV interaction for the x-intercept (**Figure 8C**) [*F*(1,30) = 14.08, *p* = 0.001] that reflected an AAV-Homer1a-induced increase in basal glutamate content in *Homer1a^-/-^* (*p* < 0.0001), but not in *Homer1a^+/+^* animals (*p* = 0.65). A comparable analysis of the slope of the linear regressions indicated that we did not replicate the genotypic difference in the extraction fraction in AAV-controls (**Figure 8D**; *p* = 0.67). Further, we did not observe any effect of AAV infusion upon this measure of glutamate release/reuptake in the NAC (Genotype × AAV: all *p*’s > 0.10). Thus, Homer1a actively regulates basal extracellular glutamate content with in the NAC without influencing release/reuptake.

## 4 Discussion

The induction of the IEG Homer isoform Homer1a is theorized to play a critical role in synaptic rearrangement and neuroplasticity relevant to various cognitive and behavioral processes, as well as pathophysiology in mental disease (cf. Shiraishi-Yamaguchi and Furuichi, 2007; Szumlinski et al., 2008a; Fagni, 2012; Marton et al., 2015). Despite its initial characterization over 20 years ago (Brakeman et al., 1997; Kato et al., 1998), we still know relatively little regarding the functional relevance of Homer1a induction *in vivo*. Herein, we employed an AAV strategy and extend prior results for *Homer1a^-/-^* mice (Park et al., 2013) by demonstrating that Homer1a within the NAC actively regulates cocaine-induced behavior, glutamate and dopamine sensitization, as well as basal extracellular glutamate levels in this region. Moreover, we demonstrate that NAC Homer1a expression actively regulates sensorimotor processing and sensorimotor-gating. We show also that Homer1a induction is necessary for normal recall of spatial memory and serves to inhibit depressive-like behavior in certain behavioral paradigms, but is not required to manifest anxiety-like behavior or cocaine-conditioned reward. Lastly, we demonstrate the importance of Homer1a induction for mGlu1/5 function within the NAC, as well as the excitability of both glutamate and dopamine terminals therein, and provide novel *in vivo* evidence that the NAC neurochemical phenotype produced by *Homer1a* deletion is distinct from that produced by deletion of the entire *Homer1* gene.

Our current knowledge concerning the behavioral relevance of distinct Homer1 isoforms can be derived from a relatively short-list of behavioral phenotyping studies of: pan-*Homer1^-/-^* mice (Szumlinski et al., 2004, 2005a; Lominac et al., 2005; Jaubert et al., 2007; Gerstein et al., 2012; Wagner et al., 2014, 2015), transgenic mice over-expressing Homer1a within the striatum (Tappe and Kuner, 2006) and transgenic mice incapable of Homer1a induction (Inoue et al., 2009; Park et al., 2013; present study). This line of interrogation is complemented and augmented by a number of reports describing the effects of site-specific manipulations of different Homer1 isoforms in the brain of wild-type rodents, as well as Homer-related mutants (Ghasemzadeh et al., 2003; Klugmann et al., 2005; Lominac et al., 2005; Szumlinski et al., 2006; Ary et al., 2013; Gerstein et al., 2013; Wagner et al., 2013, 2014, 2015; Banerjee et al., 2016; **Figure 7**). However marked procedural variations across these studies render it difficult to compare outcomes directly. This being said, the behavioral testing procedures employed in the present study of *Homer1a^-/-^* mice were highly comparable, and sometimes identical, to those used in our earlier behavioral characterization of pan-*Homer1^-/-^* mice (Szumlinski et al., 2004, 2005a; Lominac et al., 2005), facilitating interpretation of disparities and similarities in behavioral phenotypes across the two mutant lines. A side-by-side comparison of the effects of *Homer1a* versus pan-*Homer1* deletion upon spontaneous behaviors is provided in **Table 1**. As is apparent from this table and the extant literature, pan-*Homer1^-/-^* mice exhibit robust and complex behavioral anomalies (Szumlinski et al., 2004, 2005a; Lominac et al., 2005; Jaubert et al., 2007; Gerstein et al., 2012; Wagner et al., 2013, 2014, 2015). In contrast, the behavioral phenotype of *Homer1a^-/-^* mice is surprisingly similar to their wild-type controls in a variety of assays (Inoue et al., 2009; Park et al., 2013, **Table 1**), although some behavioral anomalies were observed in the present study that are discussed below.

**Table 1:** Comparison of the behavioral phenotypes observed in *Homer1a^-/-^* versus pan-*Homer1^-^****^/^****^-^* mice.

Paradigm	Dependent variable	*Homer1a^-^^/^^-^*	Pan-*Homer1^-^^/^^-^*
**Anxiety-like behavior:**			
Elevated plus-maze	Open arm entries	WT = KO^Section 3.1.1^	WT = KO^1^
	Open arm time	WT = KO^Section 3.1.1^	WT = KO^1^
Novel object test	Latency to contact	WT = KO^Section 3.1.2^	
	No. Ccontacts	WT = KO^Section 3.1.2^	WT > KO^2^
	Time in contact	WT = KO^Section 3.1.2^	WT > KO^2^
Novel environment test	Distance	WT = KO^Section 3.1.4^	WT ≤ KO^2,3^
	Latency to center	WT = KO^Section 3.1.4^
	Center entries	WT = KO^Section 3.1.4^
Habituation to Nnovel Eenvironment	Distance	WT = KO^Section 3.1.4^	WT > KO^2,3^
	Latency to center	WT = KO^Section 3.1.4^
	Center entries	WT = KO^Section 3.1.4^
**Depressive-like behavior:**			
Porsolt swim test	Latency to float	WT > KO^Section 3.1.3^	WT > KO^2,3^
	No. floats	WT = KO^Section 3.1.3^	WT > KO^2,3^
**Learning and memory:**			
Morris water maze	Training: latency to platform	WT = KO^Section 3.3^	WT < KO^1^
	Training: distance to platform	WT = KO^Section 3.3^	WT < KO^1^
	Test: time in quadrant	WT > KO^Section 3.3^	WT = KO^1^; WT > KO (rotation)^1^
**Sensorimotor processing:**			
Acoustic startle	Basal startle (0 dB)	WT > KO^Section 3.2.1^	WT = KO^1-3^
	Startle amplitude w/o prepulse	WT > KO^Section 3.2.1^; WT = KO^Section 3.2.3^	WT = KO^1-3^
PPI	74 dB prepulse	WT = KO^Section 3.2.2^	WT = KO^1-3^
	90 dB prepulse	WT > KO^Section 3.2.2^	WT = KO^1-3^

Summary of the results of comparable studies examining the effects of *Homer1a* or *Homer1* deletion (KO) upon behavior. > Denotes greater behavioral response in wild-type (WT) than KO; < denotes less behavioral response in WT than KO; = denotes no genotypic difference; ≥denotes inconsistencies in study outcomes, with reports of either no genotypic difference or greater behavioral response in WT than KO mice; ≤ denotes inconsistences in study outcomes, with reports of either no genotypic difference or less behavioral response in WT than KO. Highlighted rows indicate phenotypic similarities between *Homer1a^-/-^* and *Homer1^-/-^* mice. ^*1*^Jaubert et al. (2007); ^*2*^Szumlinski et al. (2005a); ^*3*^Lominac et al. (2005); ^*4*^Park et al. (2013).

### 4.1 Preventing Homer1a Induction Produces an Affective Phenotype That Is Distinct from That Produced by *Homer1* Deletion

Pan-*Homer1^-/-^* mice exhibit hyper-emotionality across a variety of paradigms (Szumlinski et al., 2004, 2005a; Jaubert et al., 2007; Wagner et al., 2014, 2015). In stark contrast, the anxiety-like behavior exhibited by *Homer1a^-/-^* herein was indistinguishable from that of wild-type mice, although *Homer1^+/-^* mutants did exhibit signs of *hypo*-anxiety on the elevated plus maze (**Figures 1A,B**). The present results are in line with the results of a study by Inoue et al. (2009), in which the *Homer1a* deletion did not alter anxiety-like behavior on the elevated plus-maze, open field or light–dark box tests (Inoue et al., 2009). These data for Homer1a-null mutants are interesting in light of evidence that transgenic mice over-expressing Homer1a within striatum increases anxiety signs in mice, which could be reversed upon conditional deletion of *Homer1a* (Tappe and Kuner, 2006). Taken together, the above findings from constitutive mutant mice argue that Homer1a over-expression is sufficient, but not necessary, for the manifestation of anxiety-like behavior.

However, other data in the literature argue that Homer1a induction exerts an “anxiolytic” or “anti-depressant” effect. For example, AAV-mediated Homer1a over-expression within the basolateral amygdala reduces the manifestation of fear-conditioning, as well as social interaction (Banerjee et al., 2016), while AAV-mediated restoration of Homer1a to the prefrontal cortex of pan-*Homer1^-/-^* mice reverses their hyper-anxious and depressive-like phenotype (Lominac et al., 2005). Consistent with these latter data, both *Homer1a^+/-^* and *Homer1a^-/-^* mice exhibited a reduced latency to float in the Porsolt swim test in the present study (**Figure 1E**). In fact, this particular response to a stressor was the only emotion-related phenotype expressed in common by *Homer1a^-/-^* and pan-*Homer1^-/-^* mice (see **Table 1**). Moreover, AAV-mediated Homer1a over-expression within the NAC increased the float latency in both *Homer1a^+/+^* and *Homer1a^-/-^* mice (**Figure 6C**), arguing that Homer1a induction within both the cell body and terminal regions of the corticoaccumbens projections actively gates a depressive-like phenotype – a finding in line with associations between variants in the *Homer1* gene and major depressive disorder (e.g., Rietschel et al., 2010; Strauss et al., 2012; Rao et al., 2017). However, it is noteworthy that *Homer1a* deletion did not produce a “pro-depressive” effect in our saccharin preference test. In fact, a review of the extant literature suggests that site-specific Homer1a manipulations induce more robust and consistent effects upon negative affect (Lominac et al., 2005; Tappe and Kuner, 2006; Banerjee et al., 2016) than *Homer1a* deletion (Inoue et al., 2009; **Figure 1** and **Table 1**). This raises the possibility that Homer1a induction within distinct brain regions may exert opposing roles in regulating affective responding that are masked under constitutive inhibition of IEG induction.

### 4.2 Prevention of Homer1a Induction Produces a Cognitive Phenotype That Is Distinct from That Produced by *Homer1* Deletion

Homer1a induction is theorized to facilitate the rearrangement of the synaptic architecture of excitatory glutamate synapses necessary for metaplasticity and learning (e.g., Hu et al., 2010; Fagni, 2012; Marton et al., 2015). Although associative learning is intact in pan-*Homer1^-/-^* mice (Szumlinski et al., 2004), this mutant exhibits deficits several other forms of learning and memory, including simple habituation (Lominac et al., 2005; Szumlinski et al., 2005a), spatial learning/memory (Lominac et al., 2005; Szumlinski et al., 2005a; Jaubert et al., 2007), and instrumental learning (Szumlinski et al., 2005a; Wagner et al., 2014). Moreover, pan-*Homer1^-/-^* mice are deficient in long-term depression (Ronesi and Huber, 2008) and long-term potentiation (Gerstein et al., 2012), while *Homer1a^-/-^* mice fail to exhibit homeostatic AMPA receptor scaling within cortex (Hu et al., 2010). In contrast to pan-*Homer1^-/-^* mice (Szumlinski et al., 2005a), *Homer1a*-null mutants exhibited no observable deficits in the acquisition phase of a Morris water maze (**Figure 2D**), arguing that the induction of Homer1a is not necessary for the consolidation or recall of the information pertaining to the platform location.

However, akin to pan-*Homer1^-/-^* mice (Szumlinski et al., 2005a), both *Homer1a^+/-^* and *Homer1a^-/-^* animals spent less time in the quadrant of the pool that formerly contained the hidden platform on the probe test (**Figure 2E**). While this result might reflect impaired spatial memory recall, such an interpretation is inconsistent with their intact learning of the maze location during the acquisition phase of this experiment. As no genotypic differences were observed in the amount of floating behavior during Porsolt swim testing (**Figure 1F**) or in the total distance traveled during the probe trial (**Figure 2E**), it is unlikely that *Homer1a* deletion altered swimming capacity during the memory probe test. Alternatively, the reduced time spent in the platform quadrant might reflect the deployment of an alternative platform search or escape strategy in *Homer1a* mutant mice. Indeed, pan-*Homer1^-/-^* mice employ a “chaining” strategy to successfully navigate a water-version of the radial arm maze (Lominac et al., 2005; Szumlinski et al., 2005a) and AAV-mediated restoration of Homer1c, not Homer1a, to the PFC of pan-*Homer1^-/-^* mice reverse their deficits in spatial working memory (Lominac et al., 2005). Further, hippocampal restoration of Homer1c reverses the spatial memory impairments exhibited by pan-*Homer1^-/-^* (Gerstein et al., 2012), as well as aged mice (Gerstein et al., 2013) and hippocampal over-expression of Homer1a impairs the acquisition and/or recall of spatial memories (Klugmann et al., 2005; Celikel et al., 2007). Thus, it is clear that a more concerted research effort is required to understand more precisely how Homer1a induction within specific neurocircuits contributes to spatial and non-spatial learning of relevance to the etiology and treatment of attentional, learning and memory disorders (Wells et al., 2015).

### 4.3 Preventing Homer1a Induction Profoundly Impairs *in Vivo* Regulation of NAC Neurotransmitter Levels

Since their initial discovery, the glutamatergic consequences of *Homer1* vs. *Homer1a* deletion have been studied extensively *in vitro*, with a focus on the regulation of post-synaptic signal transduction mechanisms (cf. Shiraishi-Yamaguchi and Furuichi, 2007; Worley et al., 2007; Fagni, 2012; Marton et al., 2015). However, transfection of hippocampal neuronal cultures with constitutively expressed Homer isoforms can augment, while transfection with Homer1a reduces, presynaptic neuronal activity (Sala et al., 2001) and a more recent study demonstrated opposing roles for CC- and IEG-Homer1 isoforms in regulating mGlu1/5-stimulated Ca^2+^ entry and glutamate release from astrocytes both *in vitro* and *in situ* (Buscemi et al., 2017). Further, CC- and IEG-Homer1 isoforms exert opposing effects upon mGlu1/5-dependent generation of anandamide (Jung et al., 2007), which is well-characterized to inhibit glutamate release via activation of CB1 receptors (e.g., Johnson and Lovinger, 2016). Indeed, we have reported several anomalies with respect to *in vivo* measures of glutamate function in the brains of pan*-Homer1^-/-^* mice, notably, reduced NAC and elevated PFC basal extracellular glutamate content (Szumlinski et al., 2004, 2005a), and an altered capacity of acute cocaine to elevate extracellular glutamate levels within both the NAC (increased in pan-*Homer1^-/-^*; **Table 2**) (Szumlinski et al., 2004) and PFC (decreased in pan-*Homer1^-/-^*) (Lominac et al., 2005; Szumlinski et al., 2005a) that suggest a critical role for Homer1 proteins in regulating presynaptic aspects of glutamate transmission.

**Table 2:** Comparison of the cocaine phenotype of *Homer1a^-/-^* versus pan-*Homer1^-/-^* mice.

Paradigm	Dependent variable	*Homer1a^-/-^*	Pan-*Homer1^-/-^*
**Behavior:**			
Activity monitoring	Acute: total distance	WT < KO^4,Section 3.9.2^	WT < KO^1^
	Locomotor sensitization	WT > KO^4,Section 3.9.2^	n.d.
Conditioned place-preference	Time on paired side	WT = KO^Section 3.4,3.9.3^	WT (KO1
**Neurochemistry:**			
Conventional microdialysis	Acute cocaine: dopamine	WT ≥ KO^4,Section 3.10.2^	WT = KO^1^
	Dopamine sensitization	WT > KO^4,Section 3.10.2^	n.d.
	Acute cocaine: glutamate	WT = KO^4,Section 3.10.1^	WT < KO^1^
	Glutamate sensitization	WT > KO^4,Section 3.10.1^	n.d.

*Summary of the results of comparable studies examining the effects of *Homer1a* or *Homer1* deletion (KO) upon cocaine-induced changes in behavior and neurochemistry. > Denotes greater response in wild-type (WT) than KO; < denotes less response in WT than KO; = denotes no genotypic difference; ≥ denotes inconsistencies in study outcomes, with reports of either no genotypic difference or greater response in WT than KO mice. Highlighted rows indicate phenotypic similarities between *Homer1a****^-^****^/^****^-^*** and *Homer1****^-^****^/^****^-^*** mice. ^*1*^Szumlinski et al. (2004).*

Herein, we report that, akin to *Homer2^-/-^* mice (Szumlinski et al., 2004), pan-*Homer1^-/-^* mice also exhibit a blunted capacity of the Group1 mGlu receptor agonist DHPG to stimulate glutamate release within the NAC (**Figure 4B**), which might reflect a “de-scaffolding” of mGlu1/5 receptors within neurons and/or astrocytes in this region (Brakeman et al., 1997; Tu et al., 1998, 1999; Buscemi et al., 2017). This being said, *Homer1a^-/-^* mice also exhibited blunted DHPG-stimulated glutamate release (**Figure 4A**), as well as reduced basal extracellular glutamate content within the NAC (**Figure 5B**). These results were rather unexpected given that prior transgenic work argued a more critical role for CC-Homer1 versus Homer1a expression in regulating extracellular glutamate levels *in vivo* (Lominac et al., 2005), as well as glutamate release *in vitro* (Buscemi et al., 2017) and *Homer1a^-/-^* mice exhibit *increased* cell surface expression of mGlu5 *in situ* and *in vitro* (Hu et al., 2010). While the source of the extracellular glutamate remains to be determined (i.e., neuronal, glial, or both), the capacity of DHPG to augment NAC glutamate levels in freely moving animals can be blocked by co-infusion of an mGlu1, but not mGlu5, inhibitor (Swanson et al., 2001). By extension then, the failure of both pan-*Homer1^-/-^* and *Homer1a^-/-^* mice to exhibit a DHPG-induced rise in NAC extracellular glutamate likely relates to a deficit in mGlu1-dependent, rather than mGlu5-dependent, signaling, with the DHPG phenotype of pan-*Homer1^-/-^* mice likely reflecting incapacity to induce Homer1a.

DHPG-induced glutamate release within the NAC is also activity-dependent *in vivo* (Swanson et al., 2001). However, while *Homer1* deletion does not alter the extraction fraction index of glutamate clearance/reuptake within the NAC (Szumlinski et al., 2005a), the extraction fraction was lower in *Homer1a^-/-^* mice than wild-type controls (**Figure 5C**). This difference in extraction fraction was driven primarily by genotypic differences in glutamate flux at concentrations below y = 0 (**Figure 5C**), suggesting reduced glutamate release, rather than increased glutamate reuptake in *Homer1a^-/-^* animals (Parsons and Justice, 1992). Although *Homer1a^-/-^* mice do not exhibit changes in the frequency of miniature excitatory post-synaptic potentials within cortical pyramidal neurons (Hu et al., 2010), *Homer1a^-/-^* mice exhibited robust deficits in high K^+^-stimulated release of both dopamine and glutamate within the NAC (**Figures 4C,E**), which is a finding in-line with their blunted dopamine response to acute cocaine and their failure to exhibit both dopamine and glutamate sensitization with repeated cocaine treatment (Park et al., 2013; **Figures 8A,B**). In contrast, depolarization-induced glutamate or dopamine release was intact within the NAC of pan-*Homer1^-/-^* mice, despite their lower extracellular glutamate levels (**Figures 4D,F** and **Table 2**). This result indicates that pan-*Homer1* deletion does not grossly impair the excitability of either dopamine or glutamate terminals within this region – a finding consistent with the fact that cocaine can elevate the NAC levels of both neurotransmitters in *Homer1^-/-^* mice (Szumlinski et al., 2004). Importantly, AAV-mediated restoration of Homer1a to the NAC of *Homer1a^-/-^* mice reversed both the anomalies in basal glutamate, as well as the blunted cocaine responsiveness of dopamine and glutamate observed in *Homer1a^-/-^* animals, while NAC Homer1a over-expression in *Homer1^+/+^* mice was without any overt effect on our neurochemical measures (**Figure 8**). It remains to be determined whether or not virally mediated Homer1a expression incorporates into the post-synaptic density in a manner akin to endogenous protein to influence neurotransmission. Nevertheless, the present neurochemical data argue that Homer1a induction within the NAC actively regulates, but is not sufficient to augment, basal extracellular glutamate, as well as dopamine and glutamate responsiveness within this region. These data provide confirmatory evidence of a novel role for this IEG Homer1 isoform in regulating at least two neurotransmitters systems within a brain region highly implicated in incentive motivational and attentional processing.

### 4.4 NAC Homer1a Induction Actively Regulates Cocaine-Induced Behavioral Sensitization But Not Conditioned Reward

As reported previously (Park et al., 2013), the failure of *Homer1a^-/-^* mice to develop cocaine-induced neurochemical sensitization within the NAC (**Figures 8A,B**) is associated with a lack of locomotor sensitization in these animals (**Figure 7C**). Herein, we extend this prior work by demonstrating that AAV-mediated restoration of NAC Homer1a reversed the sensitization phenotype of *Homer1a^-/-^* mice, without significantly affecting spontaneous or cocaine-induced changes in locomotor activity within *Homer1a^+/+^* controls (**Figure 7**). While we previously observed no overt effects of *Homer1a* deletion upon spontaneous locomotion or acute cocaine-induced locomotor-activity (Park et al., 2013), *Homer1a^-/-^* GFP-controls exhibited blunted novelty-induced locomotion (**Figure 7A**), but greater locomotor activity in response to an acute cocaine injection (**Figure 7B**), which could relate to surgical/AAV procedures employed herein. Nevertheless, the striking parallels between the effects of manipulating Homer1a induction upon behavioral and neurochemical sensitization within the NAC observed to date argue a facilitatory role for Homer1a induction in the development of dopamine/glutamate plasticity that sensitizes drug-induced psychomotor activity.

The capacity of cocaine to augment NAC Homer1a expression (Brakeman et al., 1997; Ghasemzadeh et al., 2009b; Park et al., 2013) is reported to develop tolerance with repeated drug experience (Ghasemzadeh et al., 2009b). However, repeated cocaine exposure also reduces the NAC expression of constitutively expressed Homer1 and Homer2 proteins (e.g., Swanson et al., 2001; Ary and Szumlinski, 2007; Ben-Shahar et al., 2009; Ghasemzadeh et al., 2009a, 2011; Knackstedt et al., 2010; Loweth et al., 2014). A cocaine-induced reduction in the relative expression of CC-Homers is predicted to bias intracellular signaling within the NAC in favor of the dominant negative actions of the IEG isoforms, which we have now confirmed, herein, to be necessary for the development of drug-sensitized behavior. Consistent with this notion, pan-*Homer1* deletion (Szumlinski et al., 2004; Lominac et al., 2005) or anti-sense oligonucleotide-mediated knock-down of Homer1 expression within the NAC augments the acute locomotor-stimulatory effects of cocaine (Ghasemzadeh et al., 2003). Conversely, restoration of the CC-Homer2b isoform to the NAC of cocaine *Homer2^-/-^* mice (Szumlinski et al., 2004) or over-expression of either Homer1c or Homer2b within the NAC of cocaine-sensitized rats (Szumlinski et al., 2006) prevents their sensitized locomotor and glutamate responses to cocaine. Given the association between polymorphisms in the human *Homer1* with disorders characterized by preservative behaviors (e.g., addiction, autism, and schizophrenia) (Norton et al., 2003; Dahl et al., 2005; Kelleher et al., 2012), it will be important to understand more precisely the mechanisms through which Homer1a modulates the capacity of cocaine, as well as other psychotomimetic drugs, to induce neuroplasticity within corticostriatal circuits that underpin enduring psychomotor hyperactivity.

Our prior studies of pan-*Homer1^-/-^* and *Homer2^-/-^* mice also demonstrated active and necessary roles for CC-Homer isoform expression within the NAC in regulating more complex forms of drug-induced associative and instrumental learning (Szumlinski et al., 2004, 2005b, 2008b; Lominac et al., 2005; **Table 3**). Indeed, disruption of Homer1-dependent binding within the NAC core subregion prevents both cue- and cocaine-primed reinstatement of drug-seeking (Wang et al., 2013), although AAV-mediated over-expression of either Homer1c or Homer2b within this subregion does not prevent the incubation of cue-elicited cocaine-seeking in rat models of drug self-administration (Loweth et al., 2014). Given the robust effects of *Homer1a* deletion upon cocaine-induced behavioral and neurochemical sensitization within the NAC, we were very surprised by the absolute lack of genotypic differences upon the capacity of cocaine to elicit a conditioned place-preference in mice (**Figures 3**, **7D**). The cocaine doses and place-conditioning procedures employed in this study are sensitive to the effects of other transgenic manipulations of Homer and/or mGlu5 function (Szumlinski et al., 2004; Ary et al., 2013; Park et al., 2013). In fact, the dose-response function for cocaine-induced place-conditioning in both pan-*Homer1^-/-^*, as well as *Homer2^-/-^*, mice is shifted to the left of wild-type controls, indicating that deletion of either CC-Homer isoform increases behavioral sensitivity to cocaine-conditioned reward (Szumlinski et al., 2004). Important for data interpretation, the negative results for place-conditioning were derived from the same *Homer1a^+/+^* and *Homer1a^-/-^* that exhibit blunted cocaine-induced behavioral and neurochemical sensitization (AAV-naïve, Park et al., 2013; AAV-infused, **Figures 7**, **8**) and thus, the disparate findings across the different dependent measures cannot reflect cohort effects.

**Table 3:** Comparison of the NAC neurochemical phenotype of *Homer1a^-/-^* versus pan-*Homer1^-/-^* mice.

Paradigm	Dependent variable	*Homer1a^-/-^*	Pan-*Homer1^-/-^*
Glutamate no net-flux	x-intercept (basal glutamate)	WT > KO^Section 3.7,3.10.3^	WT > KO^1^
	Slope (release/reuptake)	WT ≥ KO^Section 3.7,3.10.3^	WT = KO^1^
Reverse dialysis	DHPG-stimulated glutamate release	WT > KO^Section 3.5^	WT > KO^Section 3.5^
	K^+^-stimulated glutamate release	WT > KO^Section 3.6^	WT = KO^Section 3.6^
	K(-stimulated dopamine release	WT > KO^Section 3.6^	WT = KO^Section 3.6^

*Summary of the results of comparable studies examining the effects of *Homer1a* or *Homer1* deletion (KO) upon *in vivo* measures of basal glutamate function. > Denotes greater response in wild-type (WT) than KO; < denotes less response in WT than KO; = denotes no genotypic difference; ≥ denotes inconsistencies in study outcomes, with reports of either no genotypic difference or greater response in WT than KO mice. Highlighted rows indicate phenotypic similarities between *Homer1a****^-^****^/^****^-^*** and *Homer1****^-^****^/^****^-^*** mice. ^*1*^Szumlinski et al. (2005a); ^*2*^Szumlinski et al. (2004).*

Although both transgenic and pharmacological evidence indicates that a cocaine-conditioned place-preference can develop independent of a dopamine-sensitized state within the NAC (e.g., Szumlinski et al., 2004, 2007), in our experience and to the best of our knowledge, the *Homer1a^-/-^* mouse is the first example in which the magnitude of drug-conditioned reward in mice is independent of a glutamate-sensitized state within the NAC (e.g., Szumlinski et al., 2005a,b, 2008b, 2017; Penzner et al., 2008). Thus, while increasing NAC glutamate is sufficient to augment the magnitude of drug-induced place-conditioning (e.g., Szumlinski et al., 2017), NAC glutamate hypersensitivity does not appear to be required for this form of drug-related learning. Whether or not *Homer1a* deletion impacts more complex drug-related instrumental learning remains to be determined.

The NAC receives major glutamate input from the PFC and local manipulations of both CC- and IEG-Homer expression can regulate extracellular glutamate, as well as the cocaine responsiveness of glutamate within the PFC (Lominac et al., 2005; Szumlinski et al., 2005a; Ary et al., 2013). In fact, mimicking cocaine-induced changes in the expression of different Homer isoforms within PFC (Ary and Szumlinski, 2007; Ben-Shahar et al., 2009; Gould et al., 2015), by increasing Homer2 expression or reducing Homer1c expression, produces a number of glutamatergic adaptations within the NAC of cocaine-naïve mice that resemble those observed in cocaine-experience rodents (Ary et al., 2013). Importantly, mimicking the cocaine-induced imbalance in CC-Homer isoforms within PFC is also sufficient to potentiate a cocaine-conditioned place-preference (Ary et al., 2013). Conversely, Homer1c over-expression within ventral PFC prevents cocaine-primed reinstatement in a rat self-administration model (Gould et al., 2015). As withdrawal from repeated cocaine augments the glutamate responsiveness to drug-associated cues (Shin et al., 2016) and prior work indicates that *Homer1a* induction is required for normal homeostatic scaling of glutamatergic transmission within cortex (Hu et al., 2010), it will be important in future work to characterize the importance of Homer1a induction for the gating of corticofugal glutamate output from PFC subregions and the executive control the PFC exerts over subcortical structures regulating incentive motivation, affect and learning. It is clear that Homer1a function is complex, strengthening or weakening synaptic connectivity in manner that depends upon on-ongoing cellular activity and the specific intracellular signaling pathways engaged (e.g., Park et al., 2013; Marton et al., 2015). Given the interpretational difficulties associated with studying a constitutive mutant model, new animal models are required that are capable of acute up- and down-regulation of this isoform within specific neuronal and glial populations, to gain a deeper understanding of how Homer1a induction bi-directionally influences synaptic strength of relevance to the etiology of human neuropsychiatric disease.

## Author Contributions

MCD, MW, CMR, KDL, and KKS conducted the experiments. MW, KL, and KKS analyzed the data. GvJ and MK supplied the AAVs. J-HH and PFW supplied the KO mice. MCD, PFW, and KKS composed the manuscript. All authors edited the manuscript.

## Conflict of Interest Statement

The authors declare that the research was conducted in the absence of any commercial or financial relationships that could be construed as a potential conflict of interest.
